# Identification of Fiber and Asphalt Mastic Interface Failure Modes Based on the Improved Growth Curve Mixture Model

**DOI:** 10.3390/ma19173615

**Published:** 2026-08-25

**Authors:** Xunqian Xu, Tong Zhou, Wenxuan Ge, Lingyan Shan

**Affiliations:** 1 School of Transportation and Civil Engineering, Nantong University, Nantong 226019, China; xunqian_xu@ntu.edu.cn (X.X.); zhoutong_ntu@163.com (T.Z.); 2 Research Institute of Highway, Ministry of Transport, Beijing 100088, China; shanlingyan618@163.com

**Keywords:** fiber-reinforced asphalt, interfacial failure mode, cluster analysis, growth curve mixture model, multivariate power exponential distribution, PCA dimensionality reduction

## Abstract

The interface failure modes between fibers and asphalt mastic exhibit complex and diverse morphologies under the influence of multiple factors, making cluster analysis difficult. To address this issue, this study proposes an improved growth curve mixture model (IGCMM) for the unsupervised clustering of interface failure modes. Through single-fiber pull-out tests on 90 specimens under three temperatures (−10 °C, 25 °C, and 60 °C) and three fiber types (basalt, glass, and polyester), load–displacement curves were obtained. The multivariate power exponential (MPE) distribution was used to characterize the peak and heavy-tailed features of residuals. Due to the high dimensionality caused by the joint analysis of multiple datasets, principal component analysis (PCA) was employed to compress the 200-dimensional curve data into three principal components, with all model parameters estimated in the low-dimensional space. The Expectation–Maximization (EM) algorithm combined with the extended Bayesian Information Criterion (eBIC) determined the optimal eight failure mode clusters. The results exhibit a high posterior assignment confidence: 96.7% of samples had posterior probabilities exceeding 0.999. The eight statistical patterns were successfully mapped to four theoretical failure modes—fiber pull-out, medium-temperature matrix failure, high-temperature matrix failure, and mixed failure—revealing the coupled regulatory mechanism of temperature and fiber type on interface failure. The framework established in this study—“data-driven clustering–physical parameter anchoring–failure mechanism interpretation”—provides new theoretical tools and methodological support for the mesoscale interface failure diagnosis and crack resistance optimization design of fiber-reinforced asphalt pavement materials.

## 1. Introduction

Fiber-reinforced asphalt concrete is increasingly used in heavy-traffic and harsh-environment applications due to its improved crack resistance, fatigue resistance, and durability [1]. Fibers such as basalt, glass, and polyester offer multiple options for toughening asphalt concrete [2]. The reinforcing effect fundamentally depends on the stress transfer efficiency and synergistic behavior at the fiber and asphalt mastic interface [3]. Accurately characterizing the interfacial failure behavior is therefore a key prerequisite for understanding the reinforcement mechanisms and optimizing fiber selection [4].

Various methods have been developed to quantify mesoscale interfacial behavior, including molecular dynamics simulations [5,6,7], surface free energy methods [8], and discrete element modeling [9,10]. However, DSR tests reflect only the bulk viscoelastic response without capturing the single-fiber behavior [11,12], while fiber bundle pull-out tests introduce interactions that deviate from the actual fiber distribution [13]. Recently, single-fiber pull-out test platforms enable the real-time recording of complete load–displacement curves as fibers are pulled from asphalt mastic at different temperatures [14], providing a reliable tool for directly characterizing the interfacial behavior at the individual fiber scale [4,15].

However, the load–displacement data from the single-fiber pull-out tests exhibit considerable complexity [16]. Parallel specimens under identical conditions produce dissimilar curves, and curves from different conditions display diverse failure morphologies due to the temperature-induced phase transitions and differences in fiber mechanical properties. Traditional approaches—manual classification based on test conditions or a comparison of mean curves—cannot objectively capture the statistical distribution patterns or systematically identify the failure modes under multi-factor coupling. Moreover, these curves have an inherent longitudinal structure. Treating each complete curve as an individual trajectory and applying longitudinal data modeling for structured representation and unsupervised clustering offers a pathway to overcome conventional classification limitations.

Several classes of methods have been developed for clustering longitudinal data [17,18,19]. Distance-based approaches, e.g., the Fréchet distance for multidimensional functional data [20,21], are computationally simple but may be less effective in capturing curve shapes. Probability-based mixture models, such as the growth curve model (GCM), achieve parameter estimation and soft clustering via the EM algorithm [18,22,23,24]. The standard GCM assumes multivariate normal errors and a known group membership matrix [25,26], with various extensions including SITAR-d for pubertal velocity [27], latent growth curve model (LGCM) for emotional literacy dynamics [28], and applications in child weight trajectories [29]. Functional clustering methods transform discrete observations into continuous functions via basis expansion, e.g., B-spline interpolation [30], copula-based kernel mixture models [31], and the longmixr tool for high-dimensional mixed-type data [32]. Although deep-learning methods have been introduced to this domain [17,19,33], their heavy reliance on large sample sizes and their “black-box” nature limit their applicability to mesoscale experimental data.

Among the aforementioned approaches, the GCM is particularly suitable for capturing the heterogeneity of interfacial failure data because of its probabilistic characterization and structured modeling capability. However, its two key assumptions—normally distributed errors and a known group membership matrix—are often difficult to satisfy in practice. To address this, Li Rongli et al. extended the error distribution to the multivariate power exponential (MPE) distribution [34,35] and treated the group membership matrix as an unknown random variable to be estimated, thereby establishing the growth curve mixture model (GCMM) [36]. This extension enhances the model’s adaptability to heterogeneous data. Nevertheless, under high-dimensional observations, the number of covariance matrix parameters in the GCMM grows quadratically with the dimension, which can easily lead to model non-identifiability and severe overfitting.

To address the above limitation, this study establishes a data-driven framework—“statistical clustering–physical parameter anchoring–failure mechanism interpretation” for the unsupervised identification of the fiber–asphalt mastic interface failure modes. The investigation is limited to mesoscale single-fiber pull-out tests across three fiber types and three temperatures, without direct inference on the macro pavement performance. Three hypotheses underpin this framework: different physical failure mechanisms yield statistically distinct curve morphologies; the growth curve mixture model can capture such heterogeneity and provide stable probabilistic clustering; and the resulting statistical clusters can be mapped to physically meaningful failure modes via mechanical parameter calibration. Accordingly, an improved growth curve mixture model (IGCMM) integrating PCA dimensionality reduction and the MPE distribution is proposed, providing theoretical and methodological support for the mesoscale interfacial failure diagnosis and crack-resistance optimization of fiber-reinforced asphalt materials.

## 2. Methods

### 2.1. Longitudinal Data Representation of the Fiber and Asphalt Mastic Interface Failure Process

Assume that n independent sets of load–displacement curve data are obtained from experiments. All curves have been interpolated to p equally spaced displacement points {*s*_1_, *s*_2_, …, *s_p_*}, where *s_j_* = 0.01(*j* − 1) denotes the displacement value at the *j*-th point. Define the response matrix Y ∈ $R^{p\times n}$, with its *i*-th column *y_i_* = [*y_i_*_1_, *y_i_*_2_, …, *y_ip_*]*^T^* representing the load observations (in cN) of the *i*-th specimen at the *p* displacement points. Here, *y_ij_* corresponds to the interfacial pull-out force of the *i*-th specimen at displacement *s_j_*.

Define the design matrix X ∈ $R^{p\times l}$, where the *j*-th row *x_j_^T^* = [*x_j_*_1_, *x_j_*_2_, …, *x_jl_*]*^T^* gives the values of the l basis functions evaluated at displacement *s_j_*. To adequately capture the overall nonlinear geometry of the load–displacement curve—including the elastic ascending stage, the peak region, and the post-peak softening stage—this study adopts quadratic polynomial basis functions (*l* = 3):

(1) $$x_{j}^{T}\;=\;\left[1,s_{j},s_{j}^{2}\right]$$

These polynomial basis functions feature good global smoothness and analytical tractability, enabling them to capture complex curve trends during interfacial failure. It should be emphasized that *s_j_* and $s_{j}^{2}$ serve only as mathematical mappings for constructing a high-dimensional feature space. The corresponding regression coefficients represent solely the statistical weights of the curve shape and carry no independent physical constitutive dimensions. The polynomial basis functions introduced in this section, together with the growth curve mixture model presented later, have the core objective of mathematically encoding the overall geometric shape of the load–displacement curves and performing unsupervised clustering.

### 2.2. Probabilistic Generative Framework of the Growth Curve Mixture Model

#### 2.2.1. Model Assumptions and Physical Interpretation

Assume that the *n* specimens originate from r distinct failure mode classes, each corresponding to a specific growth curve. Introduce the class indicator vector zi = [z_i1_, z_i2_, …, z_ir_]*^T^*, where z_ik_ ∈ {0, 1} and $\sum_{k=1}^{r}z_{\mathrm{ik}}=\;1$. When the *i*-th specimen belongs to the k-th failure mode class, its load–displacement curve follows the growth curve model

(2) $$y_{i}=X\beta_{k}+\varepsilon_{i},z_{\mathrm{ik}}=1$$

where *β_k_* = [*β_k_*_1_, *β_k_*_2_, *β_k_*_3_]*^T^* ∈ $R^{l\times 1}$ is the curve shape coefficient vector of the *k*-th class. This vector determines the intercept position (*β_k_*_1_), the linear growth trend (*β_k_*_2_), and the degree of curvature (*β_k_*_3_) of the class-specific mean curve. Differences among the *β_k_* vectors reflect the heterogeneity in the geometric trajectories of the fiber and asphalt interface failure process, rather than directly representing any single physical constant of the material.

The random error vector εi ∈ $R^{p\times 1}$ accounts for random fluctuations and individual differences during testing. Owing to numerous stochastic factors in the interfacial failure process—such as surface defects on fibers and inhomogeneity of the asphalt mastic—the error distribution often exhibits peaked and heavy-tailed characteristics that violate the normality assumption [37]. Therefore, this study adopts the multivariate power exponential (MPE) distribution to model the errors [34,35], with the probability density function

(3) $$\begin{array}{c} f\left(\varepsilon_{i}|\Sigma ,\eta \right)=\frac{p\Gamma \left(p/2\right)}{\pi^{p/2}\Gamma \left(1+p/\left(2\eta \right)\right)2^{1+p/\left(2\eta \right)}}{\left|\Sigma \right|}^{-1/2}exp\left\{-\frac{1}{2}{\left(\varepsilon_{i}^{T}\Sigma^{-1}\varepsilon_{i}\right)}^{\eta }\right\} \end{array}$$

where Σ ∈ $R^{p\times p}$ is the covariance matrix, which captures the correlations among load observations at different displacement points. Because interfacial failure is a continuous process, the load values at adjacent displacement points are strongly correlated; hence Σ is a banded matrix. The shape parameter *η* ∈ (0, 1) controls the tail thickness of the distribution. When *η* = 1, the MPE distribution reduces to the multivariate normal distribution [38,39]. When *η* < 1, the distribution possesses heavier tails, allowing it to better accommodate the outliers and extreme load values that occur during interfacial failure.

#### 2.2.2. Derivation of the Joint and Marginal Distributions

Let the mixing proportion of the *k*-th class be *π_k_* = P(z_ik_ = 1), satisfying $\Sigma_{k=1}^{r}\pi_{k}\;=\;1$ and *π_k_* > 0. The quantity *π_k_* represents the proportion of the k-th failure mode class in the population and reflects the occurrence probability of that failure mode.

For the observed data *y_i_* of a single specimen *i*, the marginal probability density function is

(4) $$\begin{array}{c} f\left(y_{i}|\Theta \right)=\sum_{k=1}^{r}\pi_{k}f\left(y_{i}|\beta_{k},\Sigma ,\eta \right) \end{array}$$

where Θ = {*β*_1_, …, *β_r_*, Σ, ***η***, *π*_1_, …, *π_r_*} denotes all the unknown parameters of the model.

The joint log-likelihood function for all *n* independent observations is

(5) $$\begin{array}{c} logL\left(\Theta \right)=\sum_{i=1}^{n}\log\left(\sum_{k=1}^{r}\pi_{k}f\left(y_{i}|\beta_{k},\Sigma ,\eta \right)\right) \end{array}$$

Substituting the probability density function of the MPE distribution into the above expression yields

(6) $$\begin{array}{c} \begin{array}{l} \log L\left(\Theta \right)=n\log\left(\frac{p\Gamma (p/2)}{\pi^{p/2}2^{1+p/(2\eta )}}\right)-\frac{\mathrm{np}}{2}\log\Gamma \left(1+\frac{p}{2\eta }\right)-\frac{n}{2}\log|\Sigma |+\\ \sum_{i=1}^{n}\log\left(\sum_{k=1}^{r}\pi_{k}\exp\left\{-\frac{1}{2}{\left({\left(y_{i}-X\beta_{k}\right)}^{T}\Sigma^{-1}\left(y_{i}-X\beta_{k}\right)\right)}^{\eta }\right\}\right) \end{array} \end{array}$$

Because the log-likelihood function contains a logarithm of a sum, no closed-form solution can be obtained by direct differentiation. Therefore, the EM algorithm is employed for parameter estimation.

### 2.3. Parameter Estimation via the Expectation–Maximization Algorithm

**Figure 1:**
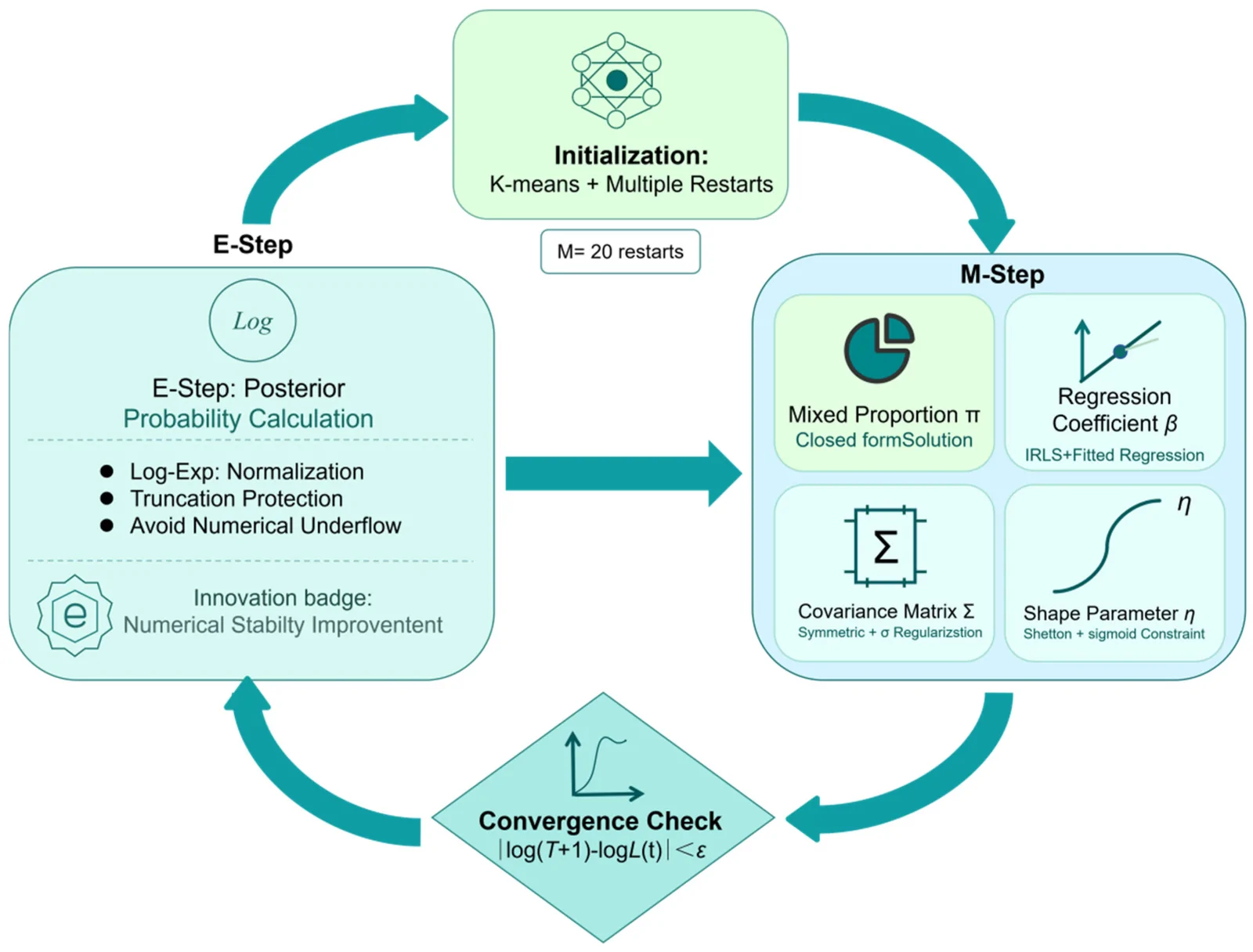
EM algorithm parameter estimation process.

To estimate the unknown parameter set Θ, of the growth curve mixture model, the Expectation–Maximization (EM) algorithm is employed [40,41], which introduces the class indicator variable *Z* as a latent variable and proceeds iteratively. The overall procedure is illustrated in Figure 1. If *Z* were treated as known, the log-likelihood of the complete data—composed of the observed data and the latent variable *Z*—can be written as Equation (7). Conversely, marginalizing over *Z* reduces the complete-data likelihood to the observed-data likelihood given in Equation (6). Substituting the probability density function of the MPE distribution into the complete-data log-likelihood yields

(7) $$\log L_{c}\left(\Theta \right)=\sum_{i=1}^{n}\sum_{k=1}^{r}z_{\mathrm{ik}}\left[\begin{array}{c} \log\pi_{k}+\log\left(\frac{p\Gamma (p/2)}{\pi^{p/2}\Gamma (1+p/(2\eta ))2^{1+p/(2\eta )}}\right)\\ -\frac{1}{2}\log|\Sigma |-\frac{1}{2}{\left({\left(y_{i}-X\beta_{k}\right)}^{T}\Sigma^{-1}\left(y_{i}-X\beta_{k}\right)\right)}^{\eta } \end{array}\right]$$

Given the parameter estimate $\Theta^{\left(t\right)}$ at the *t*-th iteration, the Q-function—the conditional expectation of the complete-data log-likelihood—is defined as

$$Q\left(\Theta |\Theta^{\left(t\right)}\right)={\;E}_{Z|Y,\Theta^{\left(t\right)}}[\log L_{c}(\Theta )]$$

#### 2.3.1. E-Step: Posterior Probability Computation

In the E-step, given the current parameter estimates $\Theta^{\left(t\right)}$, we compute the posterior probability that specimen i belongs to the *k*-th failure mode class. According to Bayes’ theorem, this posterior probability is given by

(8) $$\begin{array}{c} z_{\mathrm{ik}}^{(t+1)}=P\left(z_{\mathrm{ik}}=1|y_{i},\Theta^{\left(t\right)}\right)=\frac{\pi_{k}^{\left(t\right)}f\left(y_{i}|\beta_{k}^{\left(t\right)},\Sigma^{\left(t\right)},\eta^{\left(t\right)}\right)}{\sum_{m=1}^{r}\pi_{m}^{\left(t\right)}f\left(y_{i}|\beta_{m}^{\left(t\right)},\Sigma^{\left(t\right)},\eta^{\left(t\right)}\right)} \end{array}$$

In high-dimensional spaces, the density function values can easily overflow the representable range of double-precision floating-point numbers. To address this, we adopt the log-sum-exp normalization technique. Define

$$m_{i}=\underset{1\leq k\leq r}{\max}\left(\log\pi_{k}^{\left(t\right)}+\log f\left(y_{i}|\beta_{k}^{\left(t\right)},\Sigma^{\left(t\right)},\eta^{\left(t\right)}\right)\right)$$

The stabilized computational form of Equation (7) then becomes

(9) $$\begin{array}{c} z_{\mathrm{ik}}^{(t+1)}=\frac{\exp\left(\log\pi_{k}^{\left(t\right)}+\log f\left(y_{i}|\beta_{k}^{\left(t\right)},\Sigma^{\left(t\right)},\eta^{\left(t\right)}\right)-m_{i}\right)}{\sum_{m=1}^{r}\exp\left(\log\pi_{m}^{\left(t\right)}+\log f\left(y_{i}|\beta_{m}^{\left(t\right)},\Sigma^{\left(t\right)},\eta^{\left(t\right)}\right)-m_{i}\right)} \end{array}$$

Furthermore, a truncation protection strategy is applied to guard against numerical singularities that would cause the denominator to vanish:

(10) $$\begin{array}{c} z_{\mathrm{ik}}^{(t+1)}\;=\;\{\begin{array}{cc} \frac{\exp\left(\cdot \right)}{\sum_{m=1}^{r}\exp\left(\cdot \right)} & \mathrm{if}\sum_{m=1}^{r}\exp\left(\cdot \right)\;>\;10^{-300}\\ \frac{1}{r} & \mathrm{else} \end{array} \end{array}$$

**Theorem** **1**  (Numerical stability)**.** *After adopting Equations (9) and (10), the absolute error of the posterior probability computation is controlled at the order of* $O(\epsilon_{\mathrm{mach}})$ *(see File S1 for the proof). This guarantees robust execution of the algorithm in the high-dimensional principal component space.*

#### 2.3.2. M-Step: Parameter Updates

In the M-step, each component of the parameter set is updated sequentially by maximizing the Q-function.

(1) Mixing proportions *π_k_*. Taking the derivative of the Q-function with respect to πk and enforcing the constraint $\sum_{k=1}^{r}\pi_{k}=1$ yields the closed-form update
  (11) $$\begin{array}{c} \pi_{k}^{(t+1)}=\frac{1}{n}\sum_{i=1}^{n}z_{\mathrm{ik}}^{(t+1)} \end{array}$$
(2) Regression coefficient matrix B. For the regression coefficient vector *β_k_* of the *k*-th class, no closed-form solution exists for the normal equations. We adopt Iteratively Reweighted Least Squares (IRLS) for numerical solution [42,43]. Let the residual vector at the current iteration be $d_{\mathrm{ik}}=y_{i}\;-X\beta_{k}^{\left(t\right)}$. The IRLS weight assigned to the i-th sample for the *k*-th class is defined as
  (12) $$\begin{array}{c} w_{\mathrm{ik}}=z_{\mathrm{ik}}^{(t+1)}{\left({\left(y_{i}-X\beta_{k}^{\left(t\right)}\right)}^{T}\Sigma^{(t)-1}\left(y_{i}-X\beta_{k}^{\left(t\right)}\right)\right)}^{\eta^{\left(t\right)}-1} \end{array}$$

To prevent extreme weights from compromising numerical stability, a truncation is applied: $w_{\mathrm{ik}}\;=\;\min\left(\max\left(w_{\mathrm{ik}},10^{-10}\right),10^{6}\right)$. The update formula for *β_k_* is then

(13) $$\begin{array}{c} \beta_{k}^{(t+1)}={\left(X^{T}\Sigma^{(t)-1}X+\lambda I_{l}\right)}^{-1}X^{T}\Sigma^{(t)-1}\left(\frac{\sum_{i=1}^{n}w_{\mathrm{ik}}y_{i}}{\sum_{i=1}^{n}w_{\mathrm{ik}}}\right) \end{array}$$

where *λ* = 10^−6^ is a ridge regression regularization parameter that ensures the matrix $X^{T}\Sigma^{\left(t\right)-1}X$ remains invertible. In practice, the Cholesky decomposition is used in place of direct matrix inversion to improve numerical stability.

(3) Covariance matrix Σ. Setting the derivative of the Q-function with respect to Σ to zero gives the update for the covariance matrix. Let $S_{i}\;=\;\sum_{k=1}^{r}z_{\mathrm{ik}}^{\left(t+1\right)}d_{\mathrm{ik}}d_{\mathrm{ik}}^{T}$ be the weighted residual outer-product matrix for the i-th sample. The update is
  (14) $$\begin{array}{c} \Sigma^{(t+1)}=\frac{1}{n}\sum_{i=1}^{n}\eta^{\left(t\right)}{\left(\mathrm{tr}\left(\Sigma^{(t)-1}S_{i}\right)\right)}^{\eta^{\left(t\right)}-1}S_{i} \end{array}$$

To guarantee symmetry and positive definiteness, the following correction is applied:

(15) $$\begin{array}{c} \Sigma^{(t+1)}\;=\;\frac{1}{2}\left(\Sigma^{(t+1)}\;+\;\Sigma^{(t+1)T}\right)\;+\;\delta I_{p} \end{array}$$

where *δ* = 10^−4^ is a regularization parameter.

(4) Shape parameter *η* (the tail-thickness control parameter of the MPE distribution). No closed-form update exists for this parameter. Based on the terms involving *η* in Equation (7), an objective function g(*η*) is constructed and solved via Newton’s method, with the iterative scheme

(16) $$\begin{array}{c} \eta^{(t+1)}={\;\eta }^{\left(t\right)}-\frac{g\left(\eta^{\left(t\right)}\right)}{g^{\prime }\left(\eta^{\left(t\right)}\right)} \end{array}$$

Since the physical meaning of *η* requires it to be strictly confined to the interval (0,1), a sigmoid mapping is applied after each Newton iteration:

(17) $$\begin{array}{c} \eta^{(t+1)}\;=\;\frac{1}{1\;+\;\exp\left(-\eta^{(t+1)}\right)} \end{array}$$

This mapping guarantees that the shape parameter always lies within its valid range, regardless of the intermediate step size.

#### 2.3.3. Convergence Guarantee and Implementation Notes

The E-step and M-step are performed alternately. The iteration terminates when the change in the log-likelihood satisfies $|\log L^{\left(t+1\right)}-\log L^{\left(t\right)}|\;<\;\epsilon \left(|\log L^{\left(t\right)}|+1\right)$ with ϵ = 10^−6^. The monotonicity of the EM algorithm guarantees that the log-likelihood sequence $\left\{\log L\left(\Theta^{\left(t\right)}\right)\right\}$ is non-decreasing at each iteration (see File S1 for the proof). Hence, the algorithm is guaranteed to converge to a local maximum.

To reduce the sensitivity of the EM algorithm to initialization and avoid converging to poor local optima, a multiple-start strategy with M = 20 is adopted. In each start, a K-means clustering result initialized with a different random seed serves as the starting point. The parameter estimate that yields the highest log-likelihood is then chosen as the final output. All matrix inversions are performed using the Cholesky decomposition. Together, these numerical safeguards ensure robust performance of the algorithm on real high-dimensional data.

### 2.4. Improvement of the GCMM Through PCA Dimensionality Reduction

After interpolation, the load–displacement curves of the fiber and asphalt mastic interface have a high dimensionality (*p* = 200 in this study). The corresponding covariance matrix $\Sigma \;\in \;R^{p\times p}$ involves *p*(*p* + 1)/2 = 20,100 independent parameters, a number that far exceeds the sample size of *n* = 90. Direct fitting would therefore render the model unidentifiable and cause severe overfitting. To address this problem, we apply principal component analysis (PCA) to reduce the dimensionality of the original data. The data are projected into a low-dimensional principal component space while the dominant information is preserved. This approach circumvents the curse of dimensionality at its root.

#### 2.4.1. Consistent Projection of Response and Design Matrices in PCA

Let the original response matrix be $Y\;\in \;R^{p\times n}$. Its sample mean vector and covariance matrix are given by

(18) $$\begin{array}{c} \mu =\frac{1}{n}\sum_{i=1}^{n}y_{i}\; \end{array}$$

(19) $$\begin{array}{c} S_{Y}=\frac{1}{n-1}\left(Y-\mu 1_{n}^{T}\right){\left(Y-\mu 1_{n}\right)}^{T} \end{array}$$

where $1_{n}\;=\;{\left[1,1,\ldots ,1\right]}^{T}\;\in \;R^{n}$ is the n-dimensional all-ones vector.

The eigen decomposition of SY yields

(20) $$\begin{array}{c} S_{Y}=U\Lambda U^{T} \end{array}$$

where U = [*u*_1_, *u*_2_, …, *u_p_*] ∈ $R^{p\times p}$ is the orthogonal eigenvector matrix, Λ = diag(*λ*_1_, *λ*_2_, …, *λ_p_*) is the eigenvalue matrix, and *λ*_1_ ≥ *λ*_2_ ≥ … ≥ *λ_p_* ≥ 0. The eigenvectors corresponding to the k largest eigenvalues are selected to form the projection matrix U*_k_* = [*u*_1_, *u*_2_, …, *u_k_*] ∈ $R^{p\times k}$. The number of retained components *k* is determined by the condition that the cumulative proportion of explained variance reaches at least 0.95:

(21) $$\begin{array}{c} \frac{\sum_{j=1}^{k}\lambda_{j}}{\sum_{j=1}^{p}\lambda_{j}}\;\geq \;0.95 \end{array}$$

To preserve the linear model structure after dimensionality reduction, both response vectors yi and design matrix X must be consistently projected into the same principal component space. The projected low-dimensional response variable and design matrix are

(22) $$\begin{array}{c} y_{i}^{*}=U_{k}^{T}\left(y_{i}-\mu \right)\;\in \;R^{k\times 1} \end{array}$$

(23) $$\begin{array}{c} X^{*}=U_{k}^{T}X\;\in \;R^{k\times l} \end{array}$$

**Theorem** **2**  (Variance preservation)**.** *Under the above projection, the low-dimensional principal component space retains at least 95% of the variance of the original data, i.e.,*

(24) $$\begin{array}{c} \frac{\mathrm{tr}\left(\mathrm{Cov}\left(Y^{*}\right)\right)}{\mathrm{tr}\left(\mathrm{Cov}\left(Y\right)\right)}\;\geq \;0.95 \end{array}$$

*where* $Y^{*}\;=\;\left[y_{1}^{*},y_{2}^{*},\ldots ,y_{n}^{*}\right]\;\in \;R^{k\times n}$ *is the dimension-reduced response matrix.*

#### 2.4.2. Reparameterization of the Model After Dimensionality Reduction

In the low-dimensional principal component space, the GCMM retains its original form; only the dimensions of all parameters are reduced accordingly:

(25) $$\begin{array}{c} y_{i}^{*}\;={\;X}^{*}\beta_{k}^{*}\;+\;\varepsilon_{i}^{*},\;z_{\mathrm{ik}}\;=\;1 \end{array}$$

where $\beta_{k}^{*}\;\in \;R^{l\times 1}$ is the regression coefficient vector in the low-dimensional space, and εi* ∈ $R^{k\times 1}$ is the random error vector, which follows a k-variate MPE distribution. After dimensionality reduction, the total number of model parameters becomes

(26) $$\begin{array}{c} p_{\mathrm{total}}=lr+\frac{k(k+1)}{2}+\left(r-1\right)+1 \end{array}$$

where *lr* is the number of parameters in the regression coefficient matrix *B*∗, *k*(*k* + 1)/2 is the number of parameters in the covariance matrix Σ∗, *r* − 1 corresponds to the free parameters of the mixing proportions, and the final 1 corresponds to the MPE shape parameter *η*.

**Theorem** **3**  (Improved class separation)**.** After PCA removes the noise components associated with small eigenvalues, the class separation in the low-dimensional principal component space is no less than that in the original space. Define the class separation measures D in the original space and D* in the dimension-reduced principal component space as

(27) $$\begin{array}{c} D=\frac{2}{r(r-1)}\sum_{1<k<m}\frac{{\left\|\mu_{k}-\mu_{m}\right\|}_{2}^{2}}{\sigma_{k}^{2}+\sigma_{m}^{2}},\;D^{*}=\frac{2}{r(r-1)}\sum_{1<k<m}\frac{{\left\|\mu_{k}^{*}-\mu_{m}^{*}\right\|}_{2}^{2}}{\sigma_{k}^{*2}+\sigma_{m}^{*2}} \end{array}$$

*where μ_k_* = *Xβ_k_ is the mean curve of the k-th class in the original space, and μ_k_*_∗_ = U*k^T^μ_k_ is its projection onto the principal component space. Under the condition that the noise-related small eigenvalues λ_k_* + 1, …, *λ_p_ are discarded, D*^∗^ ≥ *D holds (see File S1 for the proof). This theorem provides a theoretical guarantee that dimensionality reduction does not weaken the separability of the cluster structure; rather, it may enhance the statistical separation between classes by eliminating noise interference.*

### 2.5. Algorithm Implementation and Convergence Analysis

#### 2.5.1. Complete Algorithmic Description

**Figure 2:**
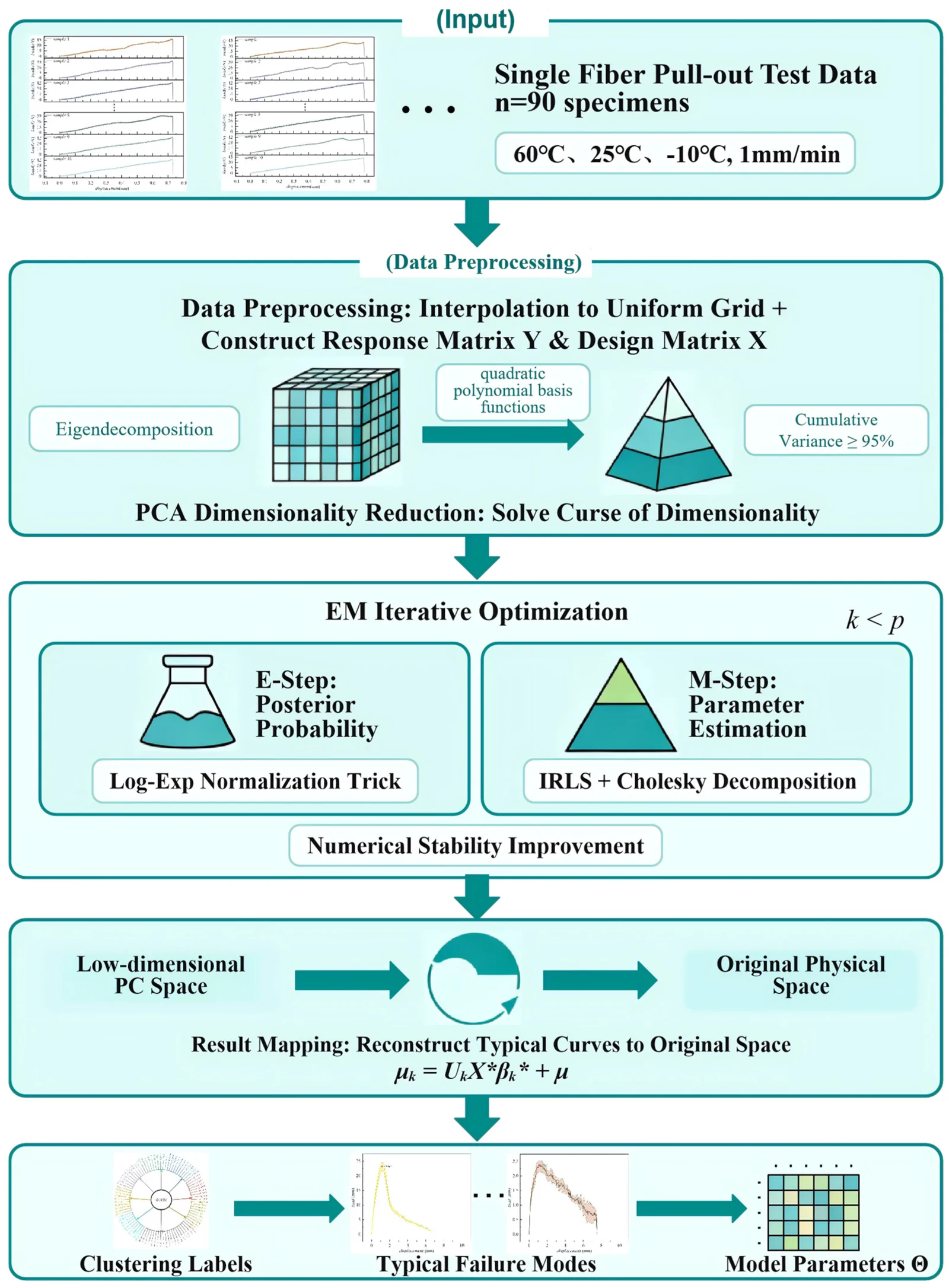
The algorithm process of the IGCMM model.

The specific modeling process of the improved mixed growth curve model is shown in Figure 2 below.

The IGCMM training algorithm uses PCA dimensionality reduction, and the specific description of the algorithm in Figure 2 is as follows.

Input: Original response matrix Y ∈ $R^{p\times n}$, design matrix X ∈ $R^{p\times l}$, number of classes r, maximum number of iterations Tmax, convergence tolerance ϵ.

Output: Model parameters Θ = {B*, Σ*, *β*, *π_k_*}, (in the low-dimensional space), cluster labels z ∈ {1, 2, …, *r*}*n*, and reconstructed typical curves in the original space *μ_k_* = U*_k_*X*β_k_* + *μ*.

Step 1: PCA dimensionality reduction.

Compute the sample mean *μ*, the covariance matrix S_Y_, and its eigendecomposition following Equations (18)–(20).

Determine the number of retained principal components k such that the cumulative explained variance reaches at least 95% via Equation (21), and form the projection matrix U*_k_*.

Obtain the dimension-reduced response matrix and design matrix using Equations (22) and (23):

$$Y^{*}\;={\;U}_{k}^{T}\left(Y-\mu 1_{n}^{T}\right),{\;X}^{*}\;=\;U_{k}^{T}X$$

Step 2: Parameter initialization.

Apply K-means clustering to the rows of Y*^T^ to obtain initial class labels z^(0)^. If any cluster is empty, re-initialize the posterior probability matrix using a Dirichlet distribution.

Initialize the regression coefficient matrix (where *Z*^(0)^ is the indicator matrix constructed from z^(0)^):

(28) $$\begin{array}{c} B^{*(0)}\;=\;{\left(X^{*T}X^{*}\;+\;\lambda I_{l}\right)}^{-1}X^{*T}Y^{*}Z^{\left(0\right)T}{\left(Z^{\left(0\right)}Z^{\left(0\right)T}\;+\;\lambda I_{r}\right)}^{-1} \end{array}$$

Initialize the covariance matrix:

(29) $$\begin{array}{c} \Sigma^{*(0)}\;=\;\frac{1}{n}\left(Y^{*}-X^{*}B^{*(0)}Z^{\left(0\right)}\right){\left(Y^{*}-X^{*}B^{*(0)}Z^{\left(0\right)}\right)}^{T}\;+\;\delta I_{k} \end{array}$$

Initialize the mixing proportions as $\pi^{\left(0\right)}\;=\;\frac{1}{n}Z^{\left(0\right)}1_{n}$ and set the shape parameter to ***η***(0) = 0.5.

Step 3: EM iteration.

For *t* = 0, 1, …, *T*_max_ − 1:

E-step: Compute the posterior probability matrix *Z*^(t+1)^ according to Equations (9) and (10).

M-step: Update the mixing proportions *π*^(t+1)^ via Equation (11); update each column of the regression coefficient matrix B*^(t+1)^ using Equation (13); update the covariance matrix $\Sigma^{*(t+1)}$ via Equations (14) and (15); update the shape parameter ***η***(t + 1) via Equations (16) and (17).

Compute the log-likelihood $\log L^{\left(t+1\right)}$.

If $|\log L^{\left(t+1\right)}-\log L^{\left(t\right)}|\;<\;\epsilon \left(|\log L^{\left(t\right)}|+1\right)$, convergence is achieved and exit the loop.

Step 4: Result transformation and output.

Reconstruct the typical curves in the original space: *μ_k_* = U_k_X*β_k_* + *μ*.

Assign cluster labels: z_i_ = argmax_1≤k≤r_ z*_ik_*

Output the model parameters Θ, the cluster labels z, and the typical curves *μ_k_*.

#### 2.5.2. Convergence Analysis

**Theorem** **4**  (Monotonicity of the EM algorithm)**.** *The EM algorithm proposed in this paper ensures that the log-likelihood function is non-decreasing [44,45,46]:*

(30) $$\begin{array}{c} \log L\left(\Theta^{(t+1)}\right)\;\geq \;\log L\left(\Theta^{\left(t\right)}\right),\;\;\;\forall t\;\geq \;0 \end{array}$$

The proof is detailed in File S1. This theorem guarantees that the objective function does not deteriorate at any iteration, serving as the theoretical cornerstone of the EM algorithm’s reliability. In practice, because the MPE distribution has heavy tails, the log-likelihood function exhibits a relatively flat curvature near the maximum, leading to a linear rate of convergence.

We adopt $|\log L^{\left(t+1\right)}-\log L^{\left(t\right)}|\;<\;10^{-6}\left(|\log L^{\left(t\right)}|+1\right)$ as the convergence criterion. Convergence is typically achieved within *T*_max_ = 500 iterations. The computational complexity per iteration is O(*rnk*^2^ + *rnl*^2^ + *nk*^3^), where the first term arises from the Mahalanobis distance calculations in the E-step, and the latter two terms arise from updating the regression coefficients and the covariance matrix in the M-step.

### 2.6. Determining the Number of Classes via eBIC

The number of classes r in the growth curve mixture model is a critical hyperparameter that governs model complexity. Too few classes lead to underfitting and fail to capture the heterogeneity of interfacial failure modes; too many classes cause overfitting and generate redundant classes with no physical meaning. We adopt the extended Bayesian Information Criterion (eBIC) to determine the optimal number of classes [47,48,49]:

(31) $$\begin{array}{c} eBIC(r)\;=\;-2\log L\left({\hat{\Theta }}_{r}\right)\;+\;\xi_{1}\log S(n,r)\;+\;\xi_{2}p_{\mathrm{total}}(r){\left(\log n\right)}^{\xi_{3}} \end{array}$$

where S(*n*,*r*) is the Stirling number of the second kind and ptotal(*r*) denotes the total number of model parameters. The logarithm of the Stirling number is approximated using the stable formula logS(n,r) ≈ nlogr−log(r!). The tuning parameters are set as ξ_1_ = 1, ξ_2_ = 0.5, and ξ_3_ = 2. This combination imposes a heavier penalty on overfitting, effectively preventing an artificial inflation of the number of classes. We evaluate eBIC over the range *r* = 2 to 12 and select the value of *r* yielding the minimum eBIC as the optimal number of classes.

## 3. Materials and Experimental Methods

A self-developed single-fiber pull-out test system was adopted to characterize the mechanical response at the fiber–asphalt mastic interface. This section details the raw materials, specimen preparation procedures, test scheme, and data preprocessing workflow.

### 3.1. Raw Materials

The asphalt mastic was prepared by mixing PG76–22 asphalt binder and limestone mineral filler at a temperature of 170 °C, with a filler-to-bitumen ratio of 1.0 by mass. The base asphalt has a viscosity of 1.8 Pa·s at 135 °C, a penetration of 71 (0.1 mm) at 25 °C, and a ductility of 48 cm at 5 °C (5 cm/min). The prepared asphalt mastic has a penetration of 34 (0.1 mm) and a ductility of 12 cm under the same test conditions.

To obtain the shear strength parameters of the asphalt mastic, temperature sweep tests were conducted on mastic samples using a dynamic shear rheometer (DSR) at a frequency of 1.0 Hz and a controlled strain of 0.1%. Based on the measured complex shear modulus, the shear strength (τ_AMS_) was derived via the viscoelastic constitutive relationship between shear strength and modulus. The results are as follows: τ_AMS_ > 2.0 MPa at −10 °C, τ_AMS_ ≥ 0.51 MPa at 25 °C, and τ_AMS_ ≈ 0.01–0.05 MPa at 60 °C.

Three types of fibers were selected: basalt fiber (BF), glass fiber (GF), and polyester fiber (PF). All fibers have a nominal diameter of 16 μm, and their basic mechanical properties are listed in Table 1.

### 3.2. Specimen Preparation and Test Procedure

**Figure 3:**
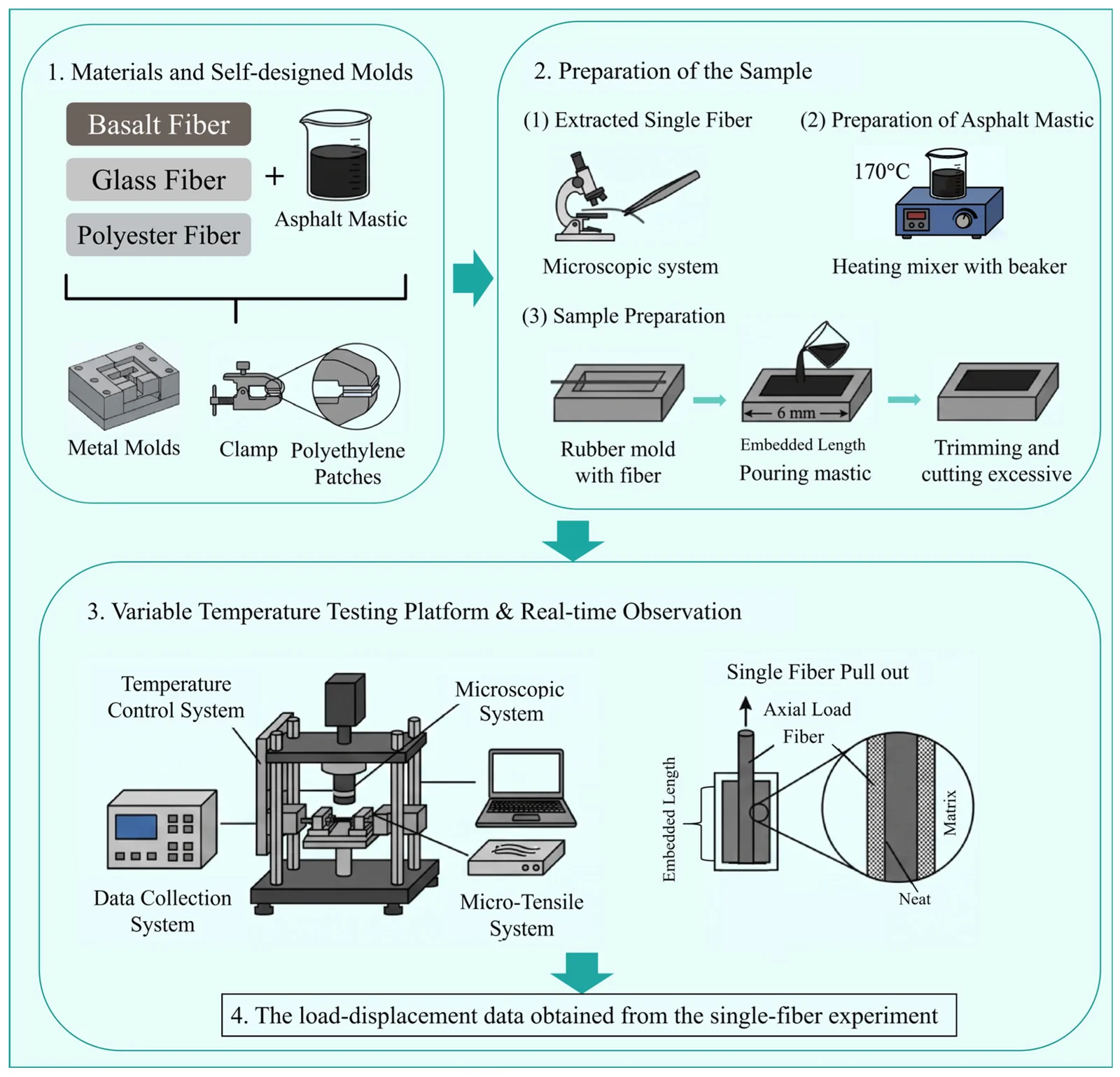
Tensile test procedure.

Nine test groups were designed with three fiber types and three test temperatures, with 10 parallel specimens per group, for a total of 90 valid specimens. The specimen preparation procedure follows the workflow shown in Figure 3, and all specimens are cylindrical asphalt mastic samples with a single fiber vertically embedded at a controlled embedment depth of 6 mm.

Self-designed combined molds were adopted for specimen molding, consisting of a metal outer mold with a central positioning hole and a polyethylene rubber inner mold, as shown in Figure 3. The central positioning hole matches the fiber diameter to ensure fiber verticality and precise control of embedment length. The complete preparation process includes three sub-steps consistent with Figure 3:

1. Single fiber extraction and fixation: Single fibers of basalt, glass, and polyester were carefully extracted from fiber bundles, respectively, to avoid surface damage or bending deformation. Each fiber was fixed through the central positioning hole of the mold, and the reserved length was adjusted to ensure an embedment depth of 6 mm inside the mold cavity.
2. Asphalt mastic preparation: Asphalt binder was preheated to 170 °C, and dried limestone mineral filler was added in batches. The mixture was stirred in a heating mixer at 3000 r/min for 15 min to achieve uniform dispersion, followed by vacuum defoaming treatment to eliminate internal bubbles. The filler-to-bitumen ratio was controlled at 1.0 by mass.
3. Specimen molding and post-treatment: The freshly prepared asphalt mastic was slowly poured into the mold with pre-fixed fibers from one side to reduce bubble entrainment. After initial cooling at room temperature, excess mastic at the top was trimmed to ensure a flat specimen surface and accurate height. Specimens were then fully cured at room temperature for 2 h, and demolded carefully with the assistance of the rubber inner mold to avoid interfacial damage.

To prevent mineral filler settlement and ensure mastic homogeneity around the embedded fiber, three control measures were implemented:

- The filler was added in batches under continuous high-speed shearing to avoid agglomeration.
- Mastic pouring was completed within 5 min after taking it out of the heating device, to shorten the high-temperature standing time.
- Specimens were cooled rapidly at room temperature immediately after pouring, to reduce the settlement time of filler particles.

The main technical challenges of specimen preparation lie in the control of fiber verticality and embedment depth, which directly affect the accuracy of interfacial shear strength calculation. In this study, a precision positioning fixture was used for auxiliary installation, and the embedment depth of each specimen was calibrated under a digital microscope before testing to ensure test reliability.

### 3.3. Single-Fiber Pull-Out Test Procedure

The single-fiber pull-out tests were performed on a self-developed micro-tensile platform corresponding to the test setup shown in Figure 3. Test temperatures were set at −10 °C, 25 °C, and 60 °C, covering typical service conditions of asphalt pavement, and the loading rate was kept constant at 1 mm/min.

Before testing, all specimens were conditioned in the environmental chamber at the target temperature for 24 h to ensure uniform temperature distribution. During testing, the free end of the fiber was clamped by a precision fixture, and load and displacement signals were synchronously collected in a time-triggered mode at a sampling frequency of 100 Hz.

### 3.4. Data Preprocessing

Raw load–displacement signals were output by the data acquisition system, as shown in the last module of Figure 3. To eliminate high-frequency test noise, unify data dimensions, and support subsequent longitudinal data modeling, two-step preprocessing was performed on all curves:

(1) Equal-displacement resampling: Under the constant loading rate of 1 mm/min, the time-series raw data have high sampling density and redundant data points. The raw curves were first resampled into an equal-displacement-interval sequence with a step size of 0.005 mm within the full range of 0–10 mm. This step yielded approximately 2000 valid data points per specimen, which completely retained the core morphological features of the curves while reducing data redundancy. The upper limit of 10 mm was determined because the maximum actual pull-out displacement of all valid specimens was less than 10 mm, ensuring full coverage of the complete failure process of each specimen.
(2) Uniform interpolation for modeling: To further standardize the input dimension of the clustering model, all resampled curves were secondarily interpolated into 200 equally spaced displacement points over the range of 0–10.0 mm via linear interpolation. For specimens whose actual pull-out displacement is less than 10 mm, the load value is set to 0 in the displacement interval beyond the actual pull-out point. This zero-value tail does not correspond to mathematical extrapolation, but reflects the physical state that the fiber is completely debonded from the mastic and the interface loses its load-bearing capacity.

## 4. Results

### 4.1. Raw Data and Preprocessing Results of Single-Fiber Pull-Out Tests

#### 4.1.1. Overall Distribution Characteristics of the Raw Test Data

**Figure 4:**
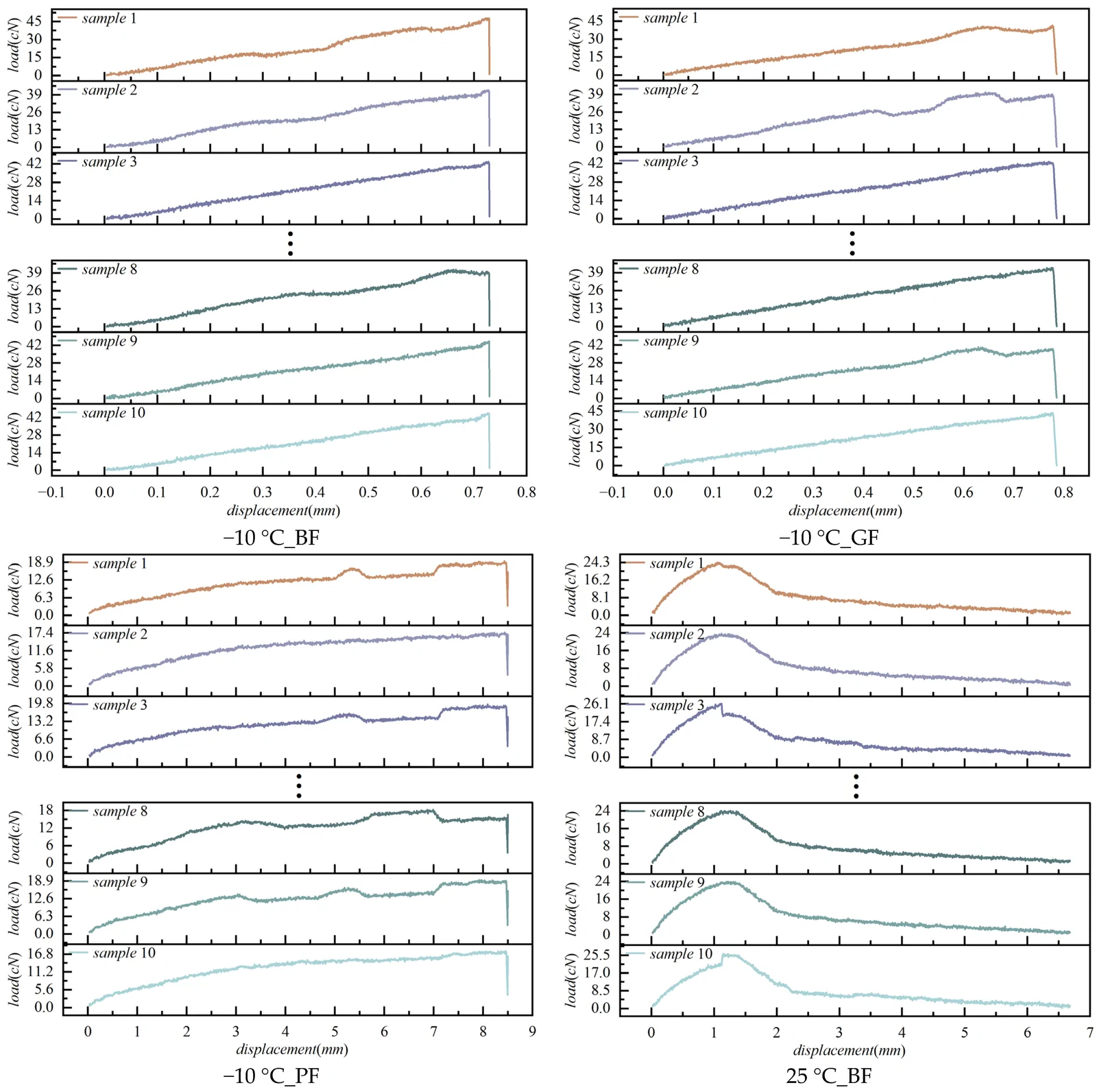

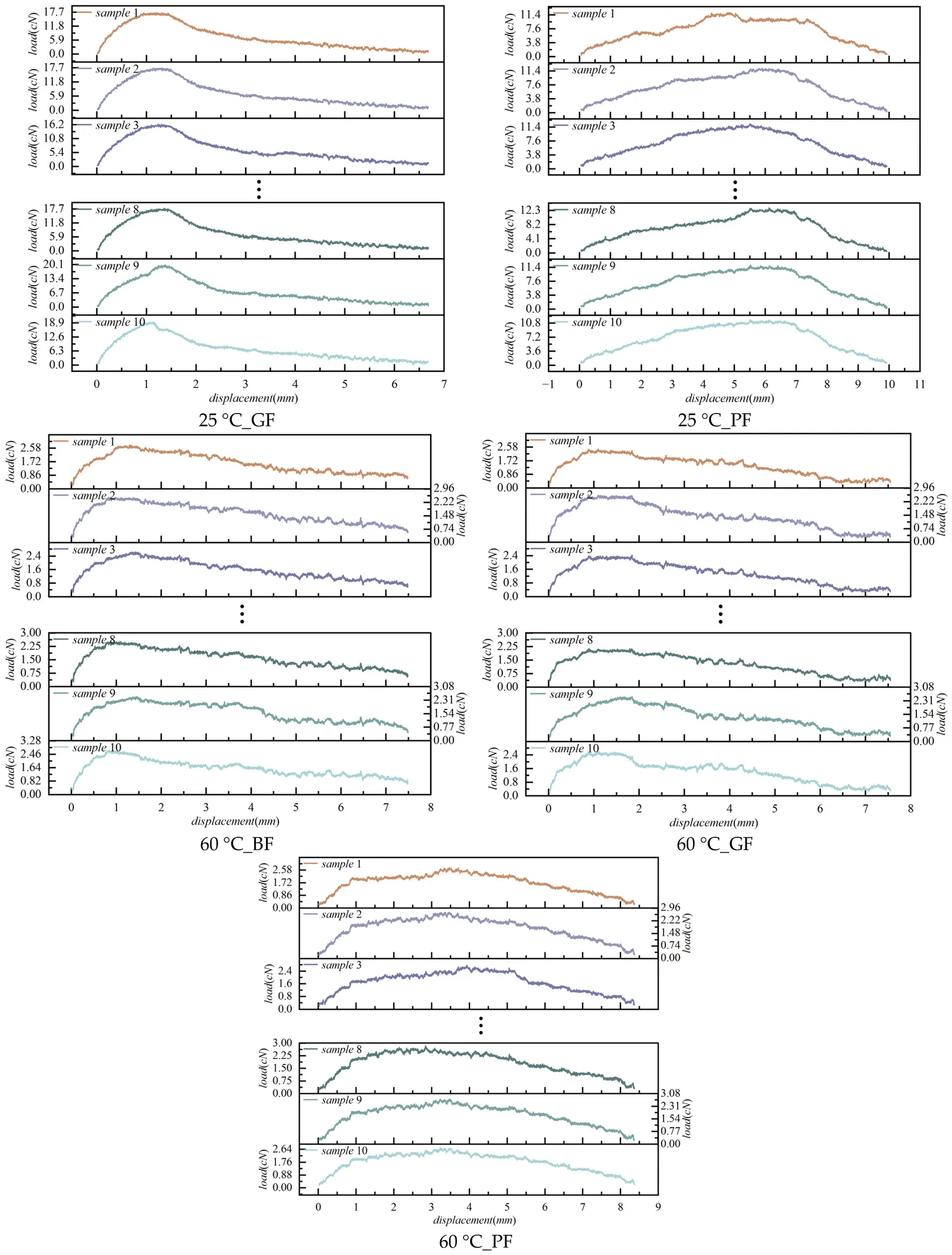
The load–displacement data obtained from the experiment.

The partial results of the raw load–displacement curves obtained from the single-fiber pull-out tests on 90 valid specimens are presented in Figure 4 and the statistical results of the indicators are shown in Table 2. These data cover three test temperatures (−10 °C, 25 °C, and 60 °C) and three fiber types (basalt fiber, BF; glass fiber, GF; and polyester fiber, PF).

The low coefficients of variation (CV < 7% for peak load, and CV < 4% for pull-out work) across all conditions confirm the satisfactory test repeatability and data quality, with no systematic differences among fiber types or temperatures.

#### 4.1.2. Validation of Data Preprocessing Effectiveness

**Figure 5:**
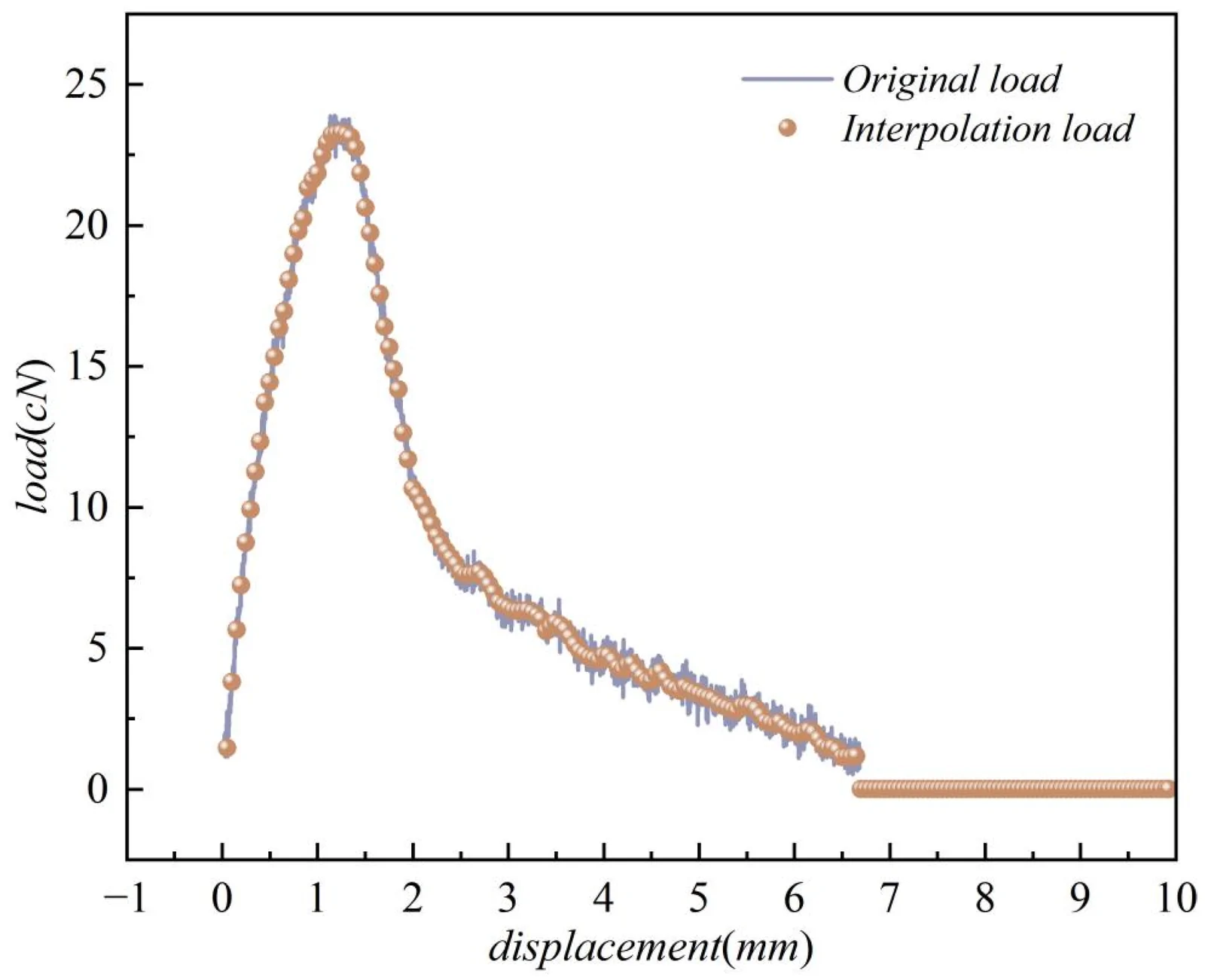
Comparison of load–displacement data before and after interpolation.

Taking Specimen 5 of basalt fiber (BF) at 25 °C as an example, the preprocessing result is shown in Figure 5. The interpolated curve effectively filters out the original high-frequency noise while preserving the core mechanical features with high fidelity: the peak load (error < 0.5%), the displacement at peak load (error ≈ 1.0%), and the overall curve shape exhibit no noticeable distortion. A uniform interpolation grid of 0–10 mm was adopted. The zero load values beyond the actual pull-out displacement of this specimen (approximately 6.7 mm) are not arbitrary mathematical extrapolations, but correspond to the physical state where the fiber is completely debonded from the asphalt mastic and the interface loses its load-bearing capacity.

Since the core discriminative information of the interfacial failure modes concentrates on the ascending section, peak region, and post-peak softening section, the zero-value tail with consistent physical boundary does not introduce spurious inter-class differences. The high classification certainty of the final clustering results further verifies the rationality of this preprocessing scheme. In the subsequent PCA dimensionality reduction and model fitting, all data points within the 0–10 mm domain are involved in the calculation, which ensures the dimensional consistency of the longitudinal data and the effectiveness of the preprocessing approach.

### 4.2. Validation of Core Assumptions of the Improved Growth Curve Mixture Model

#### 4.2.1. Validation of the Applicability of the Multivariate Power Exponential (MPE) Distribution

To verify the applicability of the multivariate power exponential (MPE) distribution described in Section 2.2.2 to the interfacial load–displacement data, we performed distribution fitting tests on the standardized residuals extracted after IGCMM fitting. The residual dataset contains 18,000 points derived from 90 specimens × 200 displacement points, with a mean of 0.0637 and a standard deviation of 1.4976. The Kolmogorov–Smirnov (KS) test was employed to compare the fits of the normal distribution and the MPE distribution, complemented by the coefficient of determination (R^2^) as a quantitative goodness-of-fit metric. The KS test statistic is defined as $D\;=\underset{x}{\sup}\left|F_{n}\left(x\right)-F(x)\right|$, where $F_{n}\left(x\right)$ is the empirical cumulative distribution function and F(x) is the theoretical cumulative distribution function. The coefficient of determination R^2^ is computed from the correlation between the fitted probability density and the empirical probability density:

$$R^{2}\;=\;1-\frac{\sum_{i=1}^{m}{\left({\hat{p}}_{i}-p_{i}\right)}^{2}}{\sum_{i=1}^{m}{\left(p_{i}-\bar{p}\right)}^{2}}$$

where $p_{i}$ denotes the empirical probability density in the i-th histogram bin, ${\hat{p}}_{i}$ the corresponding theoretical probability density, $\bar{p}$ the mean of the empirical densities, and m the number of bins. An R^2^ value closer to 1 indicates a better fit of the theoretical distribution to the data.

The Kolmogorov–Smirnov (KS) test and coefficient of determination R^2^ were adopted to evaluate the fitting performance of the two distributions. As listed in Table 3, the normal distribution has a slightly smaller KS statistic D, indicating a marginally lower global maximum deviation from the empirical distribution. However, with 18,000 residual data points, the *p*-values for both distributions are below 0.001, rejecting the null hypothesis at α = 0.05. This reflects the high sensitivity of the KS test under large sample conditions, where minor global deviations lead to hypothesis rejection, so the KS statistic alone cannot fully reflect overall fitting quality.

**Figure 6:**
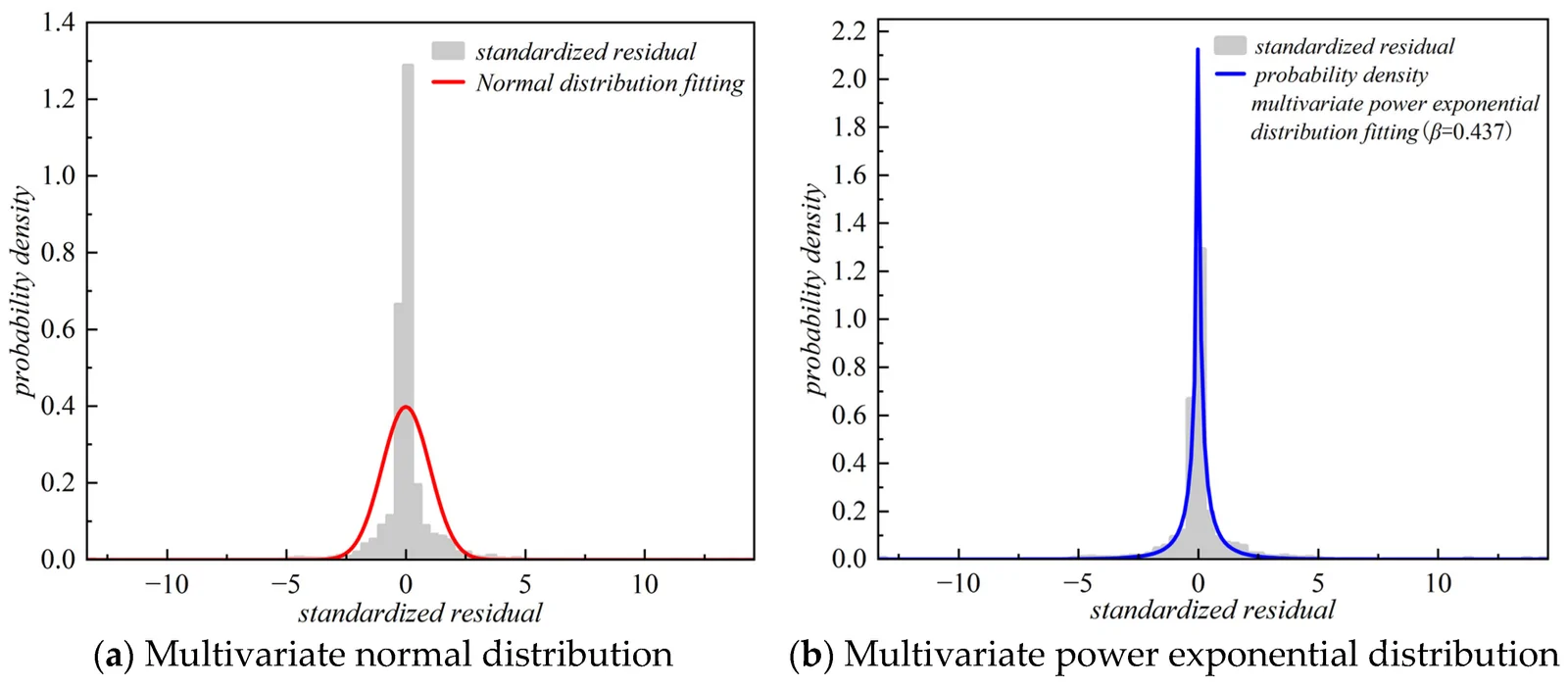
Comparison of fitting effects of different distributions.

Notably, residuals at adjacent displacement points of the same curve have an inherent serial correlation, violating the independent and identically distributed assumption of the standard KS test. Accordingly, the KS results serve only as a marginal distribution reference, while R^2^, which characterizes the overall density fitting performance, is adopted as the core evaluation index. As shown in Figure 6, the normal distribution deviates markedly from the diagonal in both tail regions of the Q-Q plot, whereas the MPE distribution scatters evenly around the diagonal with a superior tail fitting. Quantitatively, the MPE distribution yields an R^2^ of 0.9225, approximately 19.5% higher than the normal distribution. The estimated shape parameter *η* = 0.437 (<1) confirms the prominent peaked and heavy-tailed features of residuals, which are well-captured by the MPE distribution.

#### 4.2.2. Validation of PCA Dimensionality Reduction Effectiveness and Information Retention

**Figure 7:**
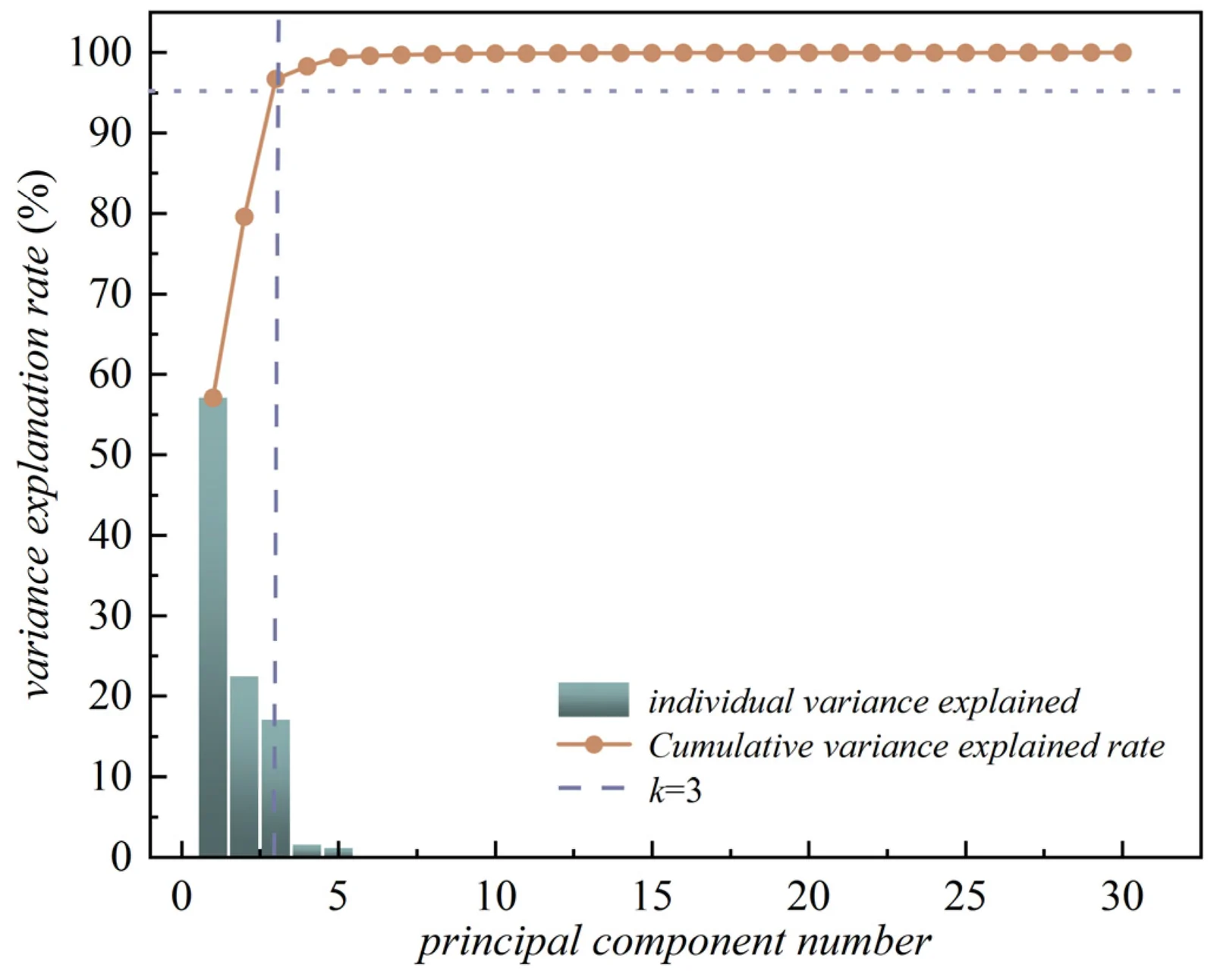
The variance explanation rates of the first 30 principal components.

Following the PCA dimensionality reduction principle described in Section 2.4.1, we apply principal component analysis to the centered 200-dimensional load–displacement response matrix $Y\;\in \;R^{200\times 90}$. The optimal number of retained principal components is determined such that the cumulative proportion of the explained variance reaches at least 95%. As shown in Figure 7, the first principal component accounts for 57.09% of the total variance, while the second and third components explain 22.52% and 17.11%, respectively. The cumulative proportion reaches 96.72%, exceeding the 95% threshold. Therefore, three principal components are retained.

**Figure 8:**
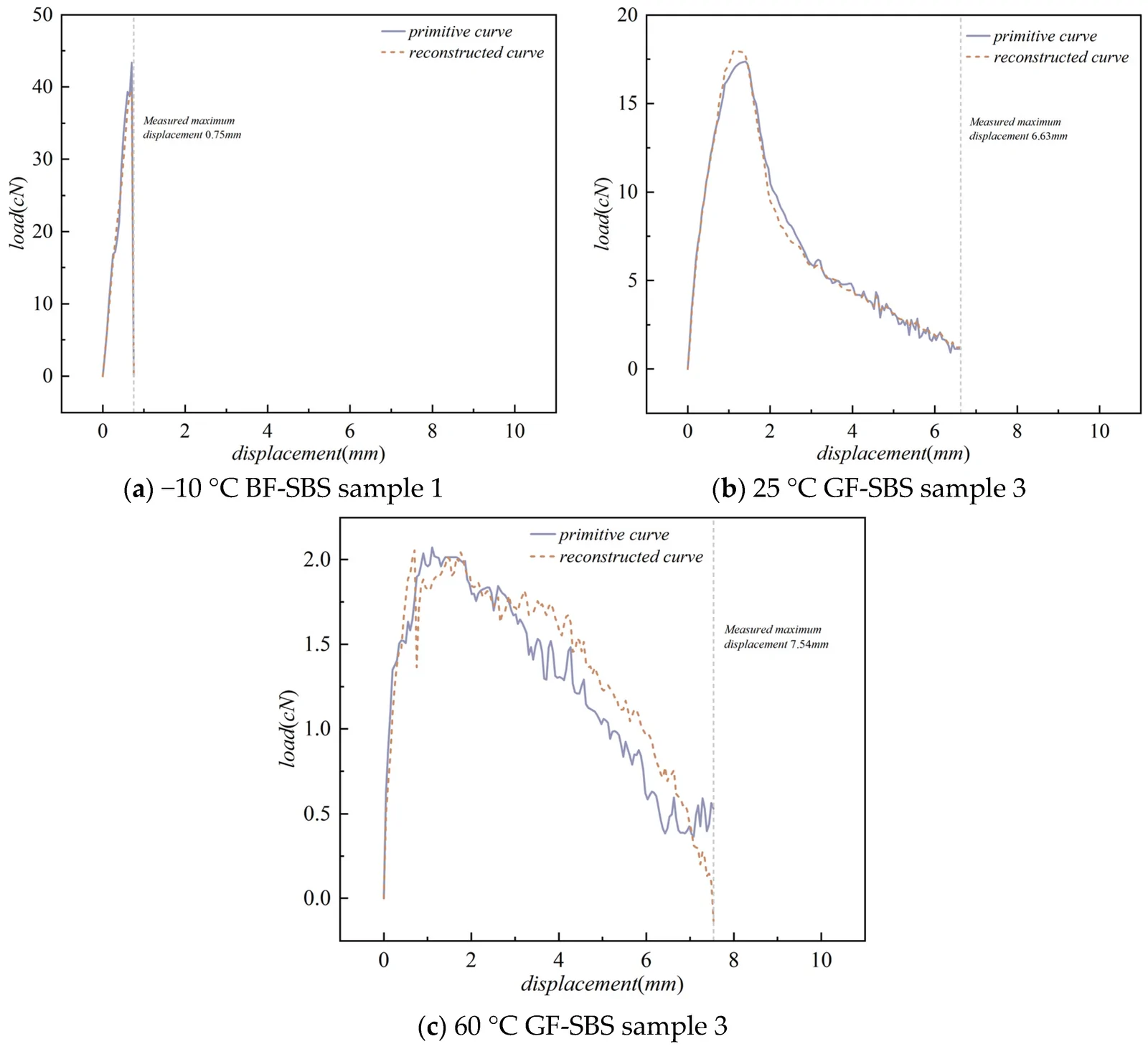
Comparison of curves after PCA dimensionality reduction.

To visually verify how well *k* = 3 preserves the core mechanical information, three representative curves—featuring low, medium, and high peak loads—are selected. Figure 8 compares the original curves with their counterparts reconstructed from the principal component space.

The reconstructed curves closely match the original ones in all key mechanical features: the elastic-stage slope, the peak position and magnitude, and the post-peak descending trend. The overall load–displacement curve morphology exhibits no noticeable distortion. The reconstruction errors are mainly confined to local fluctuations, which do not constitute critical information for the global identification of the interfacial failure modes.

Table 4 compares the reconstruction RMSE and the number of model parameters for different numbers of retained principal components. When *k* increases from 1 to 3, the reconstruction RMSE drops sharply from 3.4209 to 0.9465—a reduction of 72.3%—indicating that the first three components already capture the major variation in the data. Further increasing *k* to 10 reduces the RMSE to 0.1782, but the number of parameters grows from 38 to 87, an increase of 129%. Balancing information retention and computational cost, *k* = 3 achieves a sufficiently high reconstruction accuracy at a relatively low parameter cost. As discussed in Section 2.4, the covariance matrix in the original high-dimensional model contains *p*(*p* + 1)/2 = 20,100 parameters. After a dimensionality reduction to *k* = 3, the covariance matrix requires only *k*(*k* + 1)/2 = 6 parameters—a reduction of more than three orders of magnitude. The total number of model parameters becomes 38, which is smaller than the sample size *n* = 90. This effectively avoids the singularity and overfitting problems inherent in high-dimensional covariance matrix estimation.

### 4.3. Training Results of the IGCMM

#### 4.3.1. Optimal Number of Classes and Model Convergence

**Figure 9:**
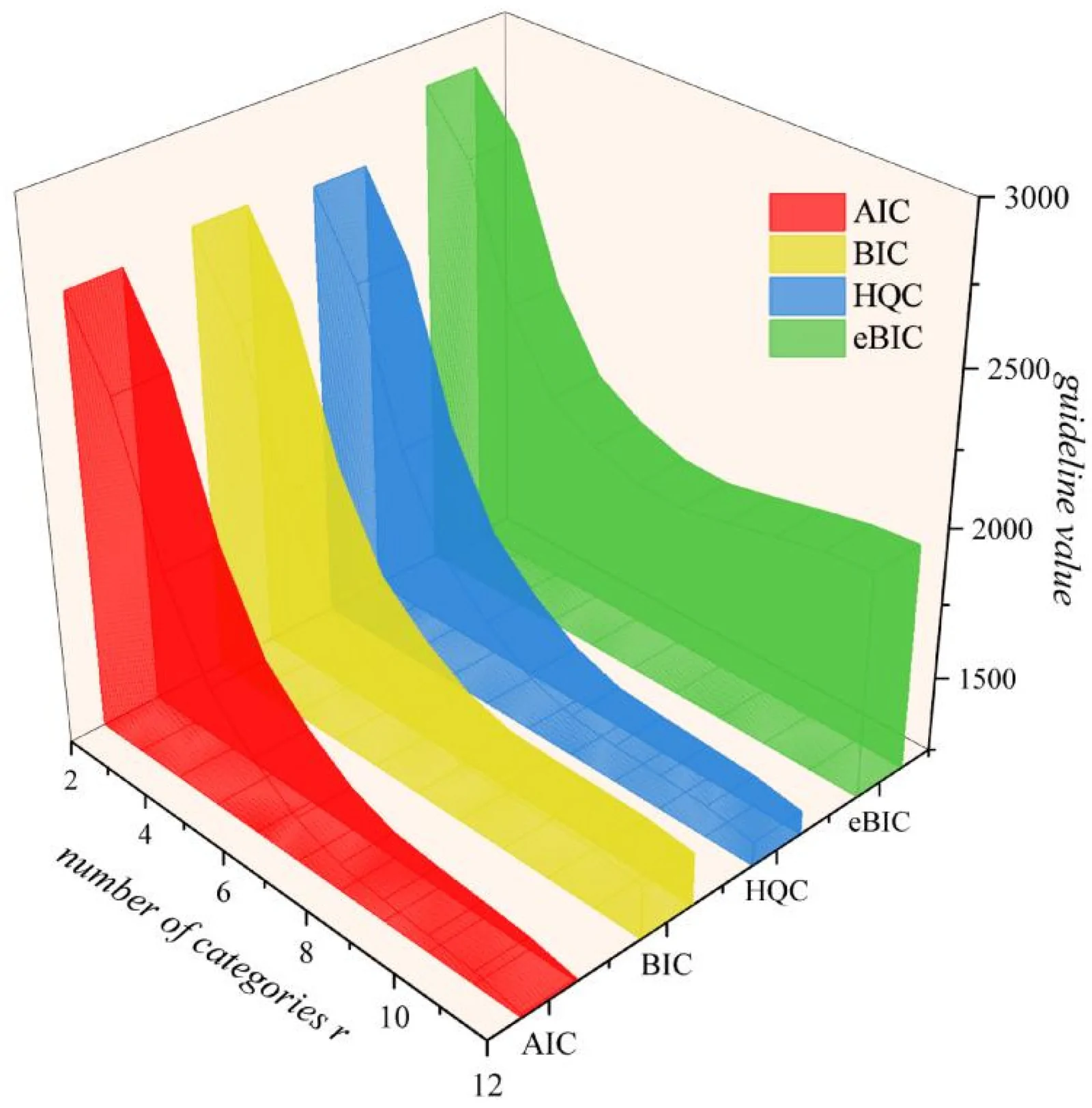
The criterion values for different categories change with the variation of *r*.

The number of classes r in the growth curve mixture model is determined using the extended Bayesian Information Criterion (eBIC). Figure 9 shows the eBIC values for *r* ranging from 2 to 12, together with the AIC, BIC, and HQC criteria for comparison. The AIC, BIC, and HQC criteria decrease monotonically across the tested range and reach their minimum at *r* = 12, as they impose weaker complexity penalties. In contrast, the eBIC with stricter regularization attains its minimum at *r* = 8 (1890.41), which is adopted as the optimal cluster number. Compared with *r* = 7 (1896.85) and *r* = 9 (1921.27), the eBIC decreases by 6.44 and 30.86, respectively, yielding a sharp and unambiguous minimum. Hence, the optimal number of classes is set to *r* = 8.

**Figure 10:**
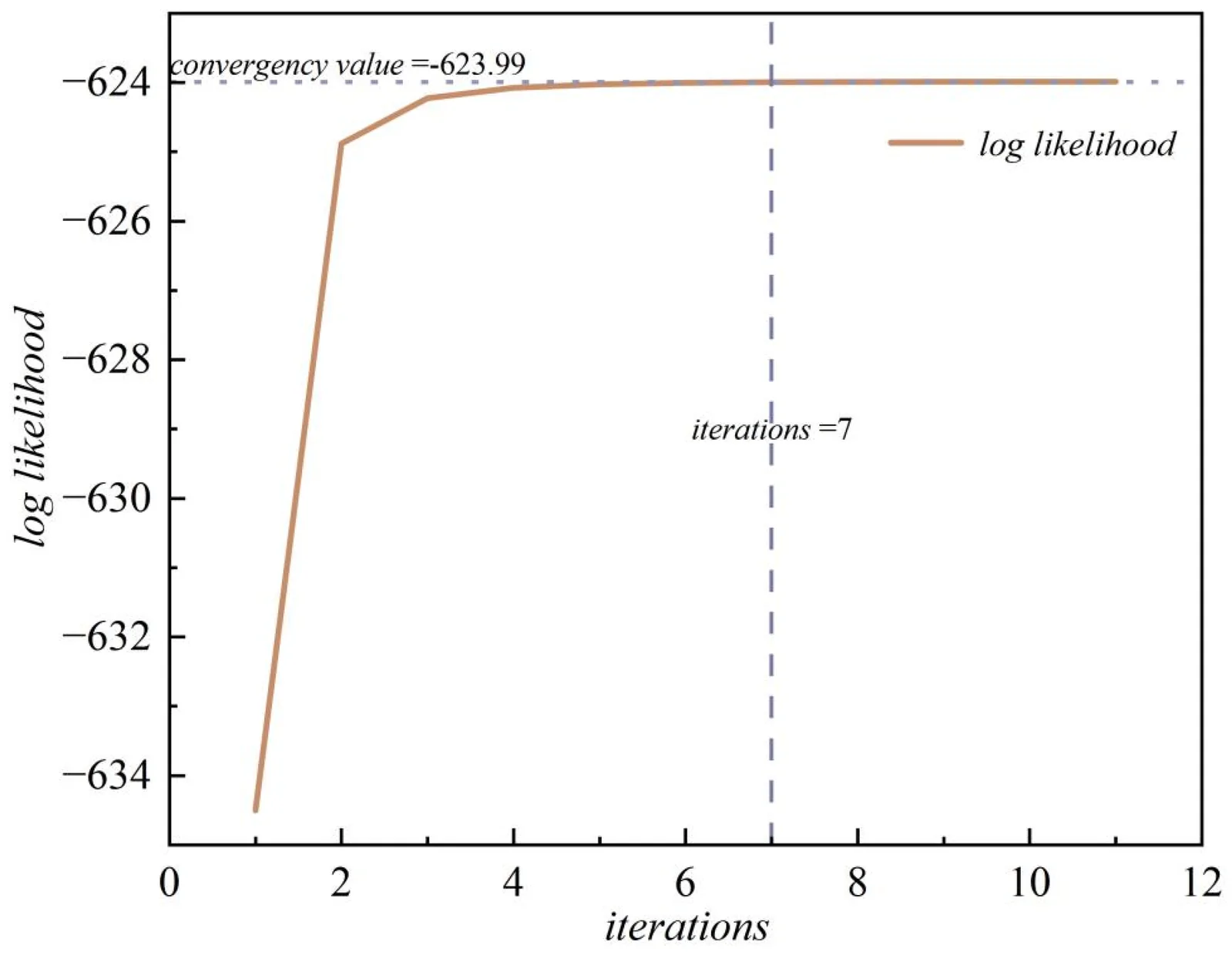
The iterative process of the EM algorithm.

With *r* = 8, the convergence behavior of the EM algorithm is illustrated in Figure 10. After the first iteration, the log-likelihood rises rapidly from its initial value to −634.50; after the second iteration, it reaches −624.88; thereafter, it gradually approaches the stable value of −623.99. Throughout the entire process, the log-likelihood increases monotonically. Statistics of the maximum posterior probability $\underset{k}{\max}\;{\hat{z}}_{\mathrm{ik}}$ for the 90 specimens are provided in File S2. The results show that 87 out of the 90 specimens achieve a maximum posterior probability exceeding 0.999, among which 82 specimens exceed 0.999. These values indicate a high internal clustering consistency and classification certainty within the model framework, reflecting the stable and unambiguous class assignment for most specimens.

The sensitivity of the EM algorithm to initialization is assessed using a 20-start strategy, where K-means is initialized with a different random seed in each start. The results are summarized in Table 5. The final log-likelihood values across the 20 starts are essentially identical (standard deviation 4.9 × 10^−12^). Using the clustering result from the first start as the reference, the adjusted Rand index (ARI) yields a mean of 1.0000. This demonstrates that twenty random initializations significantly reduce the probability of the EM algorithm getting stuck in a local optimum.

#### 4.3.2. Model Parameter Estimation Results

Table 6 summarizes the estimated regression coefficients and mixing proportions for the eight failure mode classes. The three shape coefficients—*β_k_*_1_ (intercept), *β_k_*_2_ (linear term), and *β_k_*_3_ (curvature term)—provide a complete geometric encoding of the eight failure modes from different shape dimensions. Class 4 and Class 7 exhibit a brittle response characterized by a steep rise and steep drop, with the most negative *β_k_*_2_ and the most positive *β_k_*_3_. Classes 1, 5, and 6 share a combination of a positive linear term and a negative curvature term. Class 2 and Class 8 have *β_k_*_1_≈0 and the smallest values for both *β_k_*_2_ and *β_k_*_3_, which matches the physical expectation that the interface largely loses its load-bearing capacity at high temperatures. The mixing proportions are fairly uniform: except for Class 8 (22.22%) and Class 4 (10.61%), the remaining six classes each account for 11.11%.

**Figure 11:**
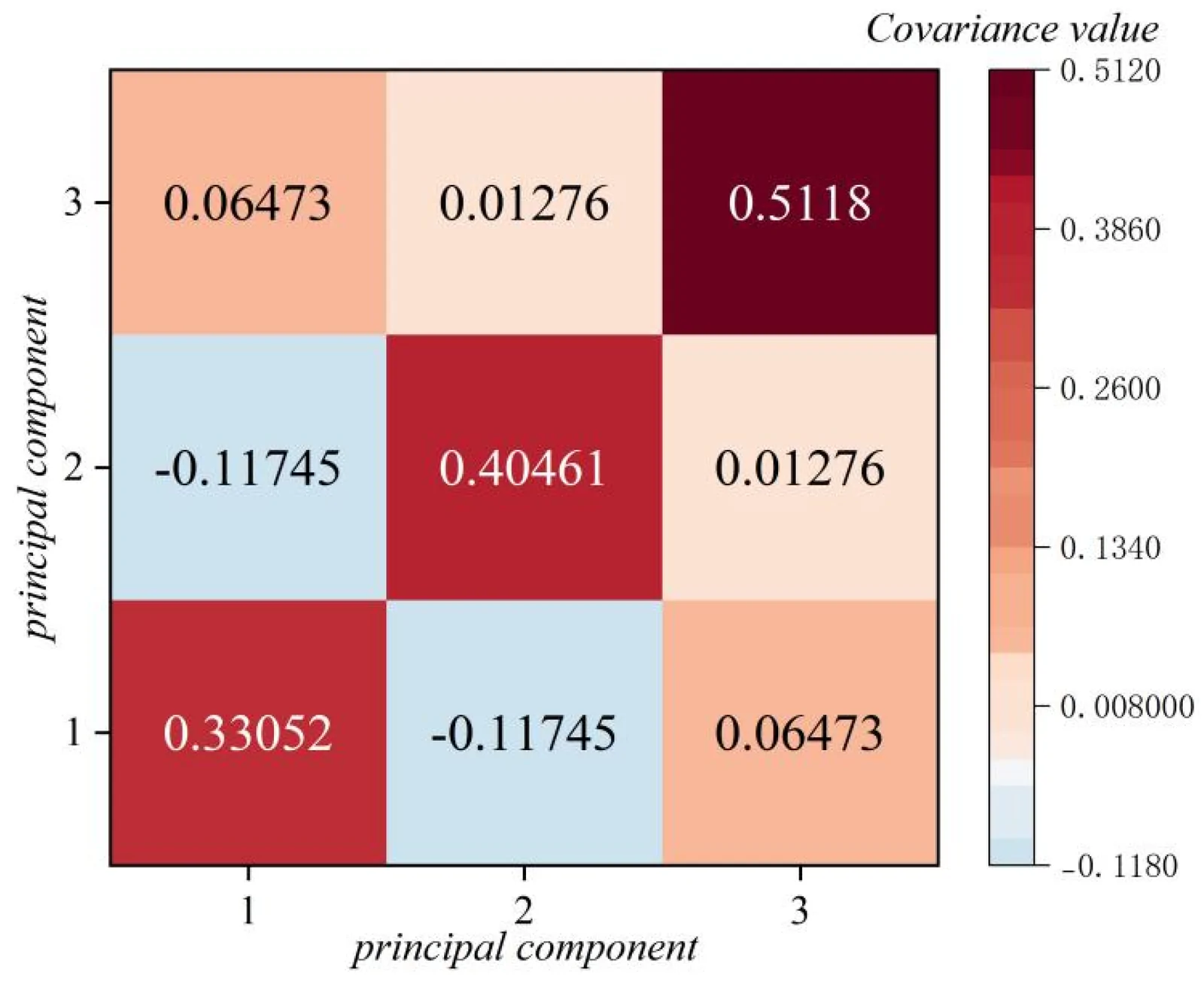
Heat map of the covariance matrix.

The estimated covariance matrix Σ∈ℝ^3×3^ is presented in Figure 11. This matrix characterizes the correlation structure among the different principal component scores; its diagonal elements (variances) reflect the data dispersion along each principal component direction.

In Figure 11, the variance in the third principal component (0.5118) is larger than those of the first (0.3305) and second (0.4046) components, indicating that the dispersion among classes is most pronounced along the third component. The absolute values of the off-diagonal elements are small, suggesting weak correlations among the principal components. This confirms that the PCA dimensionality reduction effectively removed redundant information from the original variables.

### 4.4. Interfacial Suggested Failure Mode Results

#### 4.4.1. Distribution of Clustering Results by Test Condition and Validation of Class Separation

**Figure 12:**
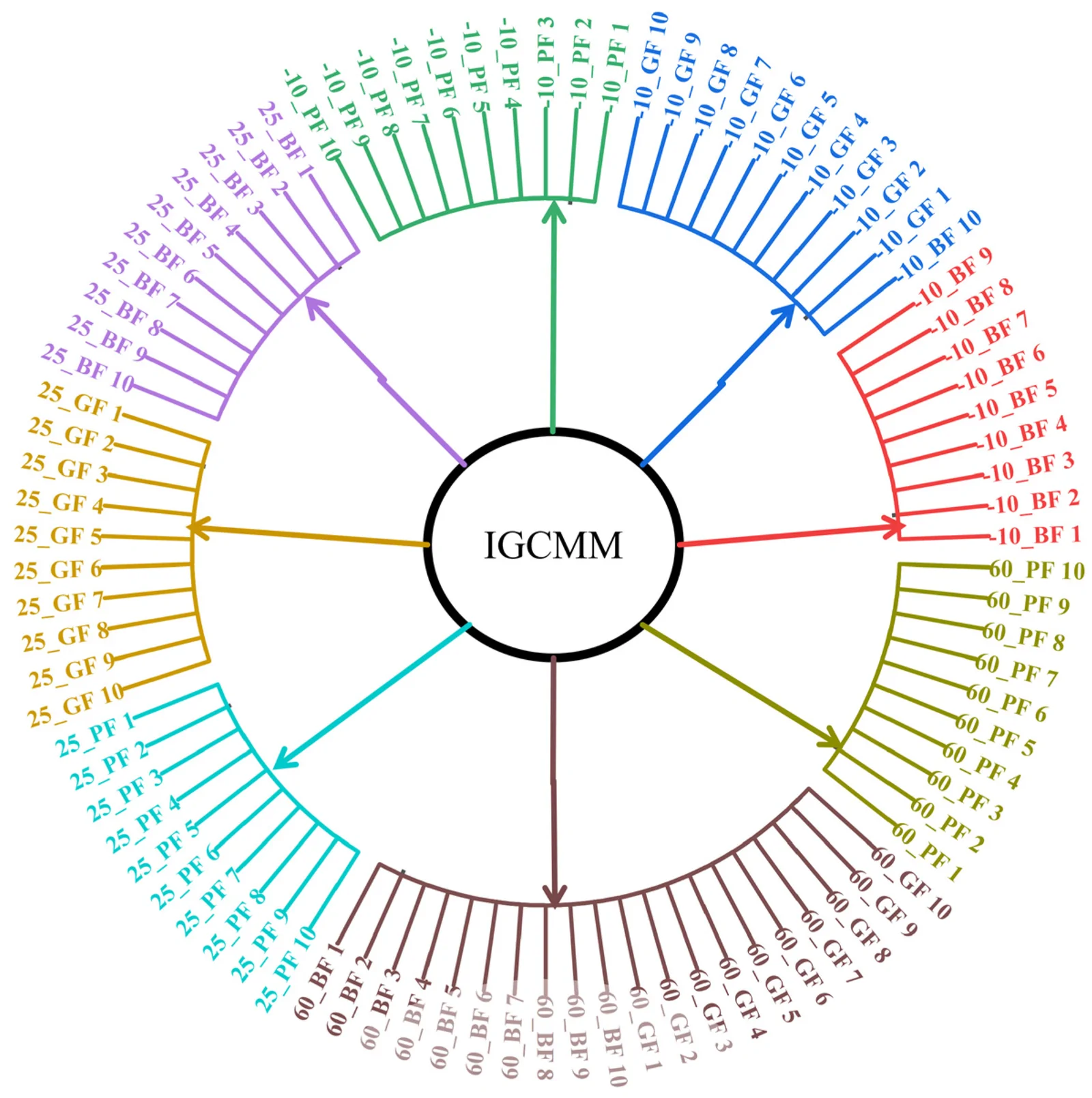
The clustering results of the failure modes of the specimens.

Figure 12 presents the distribution of cluster labels across the 90 specimens. Under each set of test conditions, all ten replicate specimens were assigned to the same class, with the sole exception of one BF specimen at −10 °C, which was grouped into Class 7. This outcome confirms the repeatability and robustness of the model-based classification. Failure modes corresponding to different test conditions exhibit a clear class separation, indicating that both the temperature and fiber type jointly govern the interface failure mode.

#### 4.4.2. Typical Load–Displacement Curve Characteristics of the Eight Failure Mode Classes

**Figure 13:**
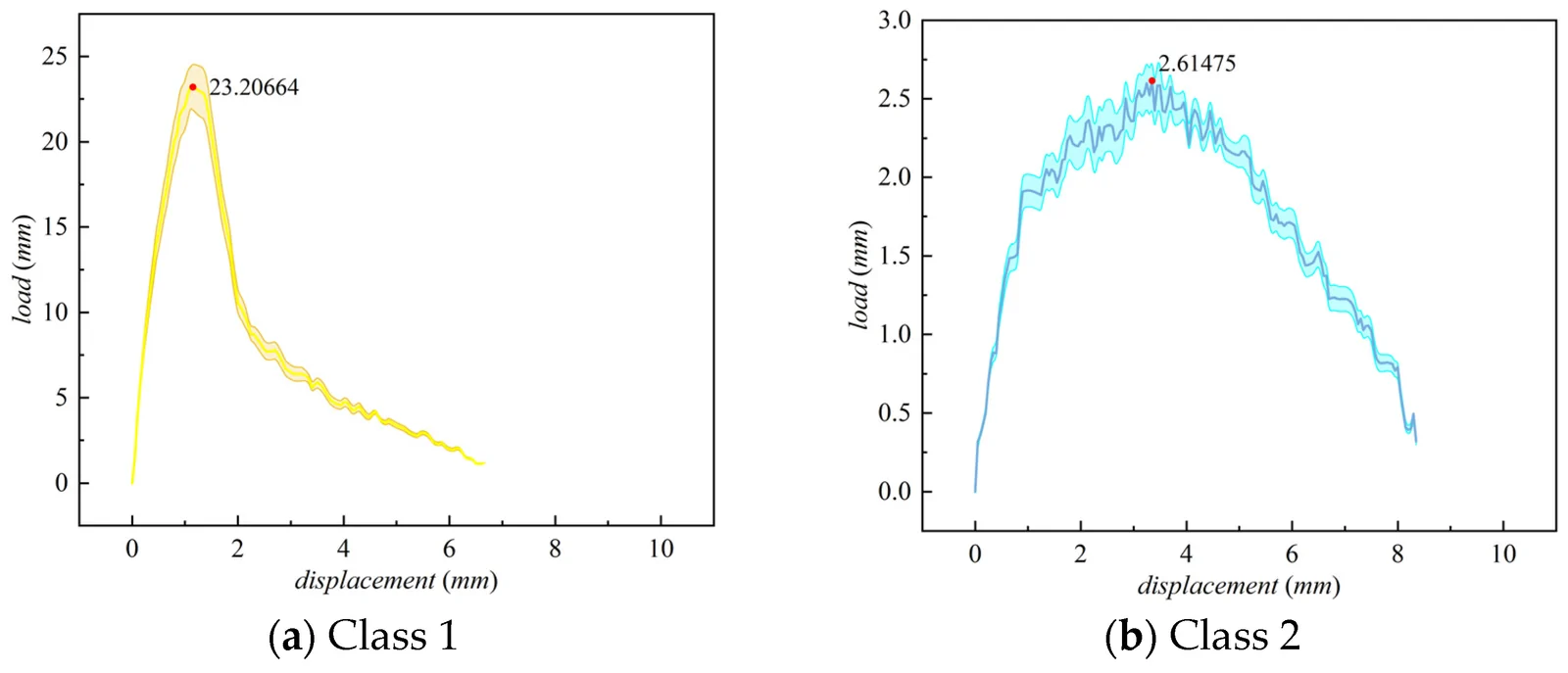

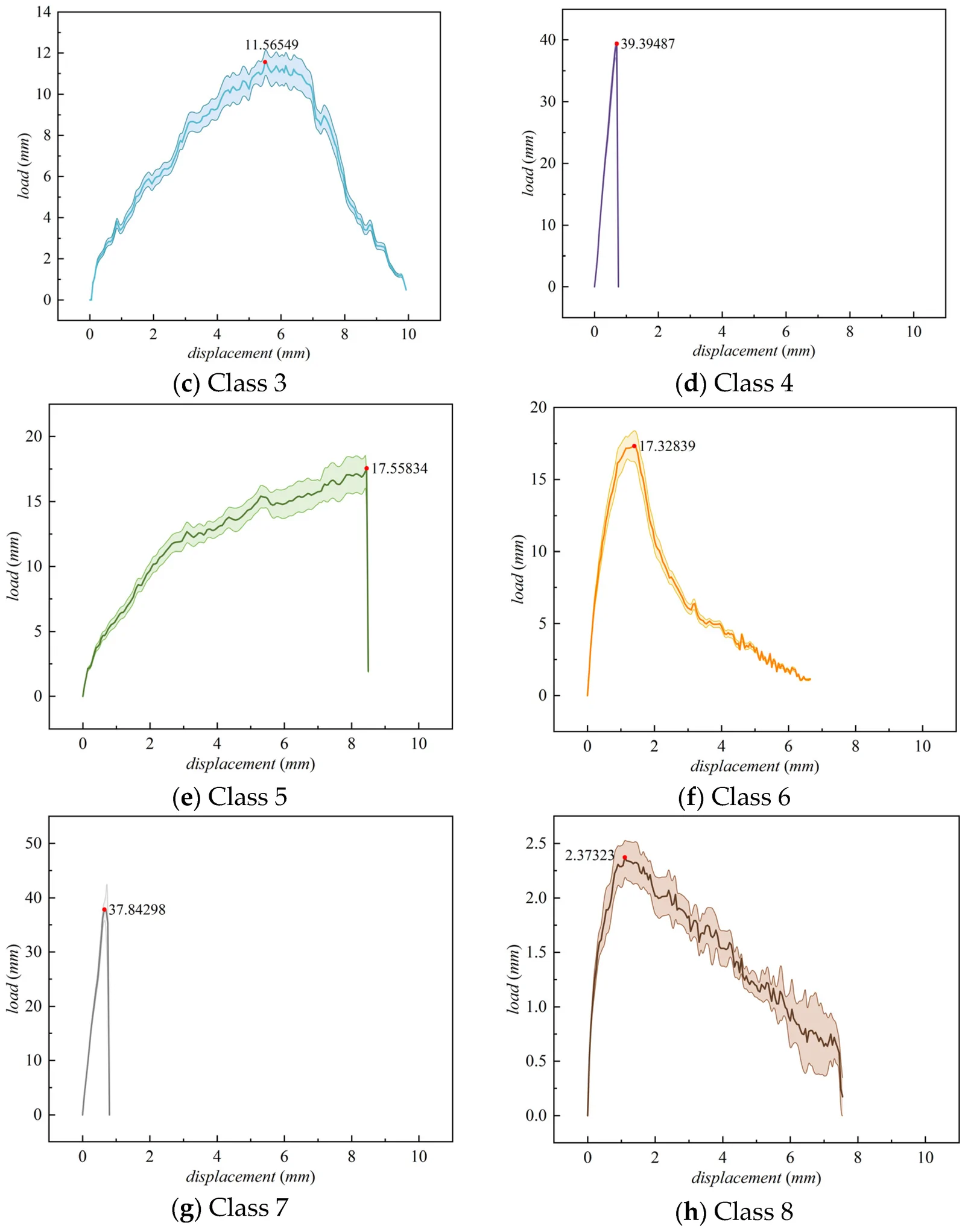
Typical load–displacement curves for eight types of failure modes.

Figure 13 presents the mean curves (solid lines) and the corresponding standard deviation bands (shaded areas) for the eight failure mode classes. Each curve reflects the average load response and its dispersion across the 200 equally spaced displacement points for the specimens belonging to that class.

Based on systematic differences in the curve morphology, the eight classes can be organized into five groups, as shown in Figure 11. Classes 4 and 7 form the first group, displaying a “steep rise–steep drop” brittle response. These classes reach the highest peak loads (approximately 38–40 cN) and their effective span does not exceed 0.75 mm. The second group comprises Classes 1 and 6, which exhibit intermediate peak loads (17.4–23.2 cN), a monotonic gradual descending trend, and an effective span of about 6.7 mm. Class 5 alone constitutes the third group, characterized by an exceptionally long, nearly horizontal plateau (2.0–8.4 mm) and the longest effective span among all classes. The fourth group is represented by Class 3, whose peak position occurs the latest (at approximately 5.5 mm) and whose curve adopts an arch-shaped long-tail morphology. Classes 2 and 8 make up the fifth group. They show extremely low load levels over the entire displacement range (peak < 3 cN) and a broad plateau with low-amplitude tailing.

These five groups display clear statistical separability across multiple morphological dimensions—including peak magnitude (2.4–40 cN, a nearly 17-fold difference), peak position (0.65–8.45 mm), ascending slope (3–60 cN/mm), and post-peak behavior (steep drop, gradual descent, plateau, or terminal steep drop). This provides a comprehensive geometric basis for the subsequent classification of interface failure modes using physical and mechanical parameters.

## 5. Discussion

### 5.1. Inferred Physical Failure Modes from Statistical Clusters

According to classical composite interface theory, the failure mode at the fiber and asphalt mastic interface is governed by the competition among the interfacial shear strength (τ_IFSS_), the fiber fracture strength (σ_FFS_), and the shear strength of the asphalt mastic (τ_AMS_). When τ_IFSS_ < τ_AMS_ and τ_IFSS_ < σ_FFS_, the interface fails by fiber pull-out. When σ_FFS_ < τ_IFSS_ and σ_FFS_ < τ_AMS_, fiber fracture occurs. When τ_AMS_ < τ_IFSS_ and τ_AMS_ < σ_FFS_, matrix failure takes place. The interfacial shear strength is calculated as

$$\tau_{\mathrm{IFSS}}=\frac{F_{\max}}{\pi \cdot D_{f}\cdot L_{f}}$$

where F_max_ is the peak load, D_f_ is the fiber diameter, and L_f_ is the embedded fiber length. A complete calculation example for Class 4 is provided as follows, with all units converted to the International System of Units:

Peak load: $F_{\max}\;=\;39.66\;\mathrm{cN}\;=\;39.66\;\times \;10^{-2}\;N\;=\;0.3966\;N$;

Fiber diameter: $D_{f}\;\;=\;16\;\mu m\;=\;16\;\times \;10^{-6}\;m$;

Embedded length: $L_{f}\;\;=\;6\;\mathrm{mm}\;=\;6\;\times \;10^{-3}\;m$;

Interfacial shear area: $A\;=\;\pi \;\cdot \;D_{f}\cdot L_{f}\approx \;3.016\;\times \;10^{-7}{\;m}^{2}$;

Interfacial shear strength: $\tau_{\mathrm{IFSS}}\;=\;\frac{F_{\max}}{A}\;=\;\frac{0.3966}{3.016\;\times \;10^{-7}}\;\approx \;1.315\;\times \;10^{6}\;\mathrm{Pa}\;=\;1.32\;\mathrm{MPa}$.

**Figure 14:**
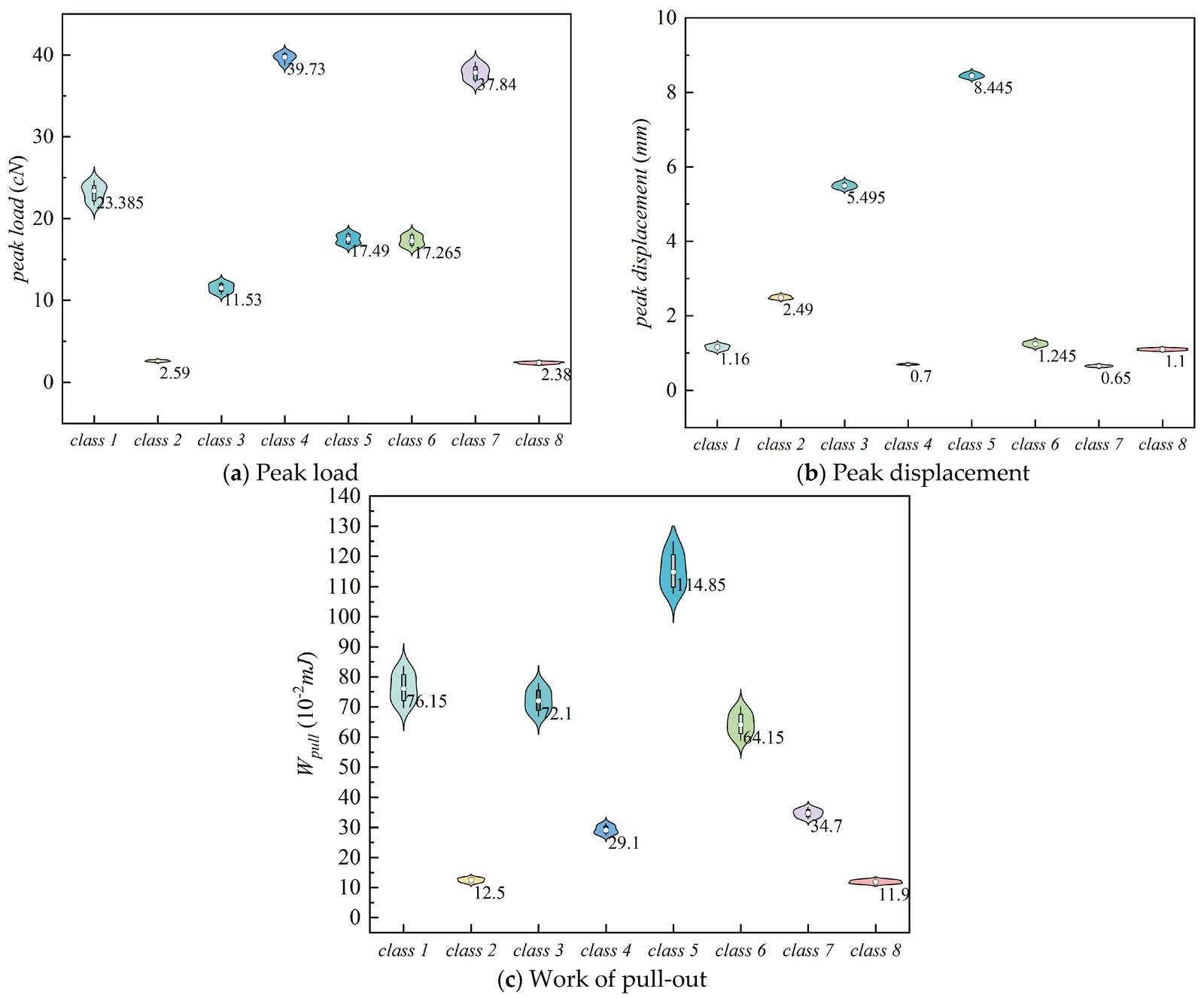
Error in physical parameters of eight-class clustering results.

Following the above method and using the typical curve data transformed back to the original space, physical parameters were computed for each of the eight cluster classes. The statistical summary of these parameters is shown in Figure 14, and the mean values together with the corresponding τ_IFSS_ values are listed in Table 7.

Several observations can be drawn from these results. The standard deviations of the three physical parameters within each class are small, and their coefficients of variation (*CV*) are all below 7%. This indicates highly consistent physical responses among specimens belonging to the same class and demonstrates the excellent repeatability of the IGCMM clustering. In contrast, the differences in physical parameters between classes are pronounced. The peak load F_max_ ranges from approximately 2.4 to 40 cN (a nearly 17-fold difference), τ_IFSS_ ranges from about 9.76 to 161.88 MPa (also nearly 17-fold), and the pull-out work W_pull_ ranges from approximately 11.9 × 10^−2^ mJ to 115.6 × 10^−2^ mJ (roughly a 10-fold difference). The clustering results exhibit significant inter-class separation across all four physical parameters. This confirms that the differences in the complete curve morphology exploited by the IGCMM can indeed be captured by the variations in a few key physical parameters.

Based on a comparison of the computed τ_IFSS_ values with the fiber fracture strength (σ_FFS_) and the asphalt mastic shear strength (τ_AMS_), the eight cluster classes are mapped to the theoretical composite interface failure modes. The mapping is presented in Table 8, and the identification criteria for each mode are as follows:

Based on the strength comparisons in Table 7, Classes 4 and 7 (−10 °C BF/GF) are inferred as fiber pull-out, since τ_IFSS_ (1.26–1.32 MPa) is far below σ_FFS_ (>2300 MPa) and below τ_AMS_ (>2.0 MPa). Classes 1, 2, 6, and 8 are identified as matrix failure, subdivided into the intermediate-temperature (Classes 1 and 6, 25 °C BF/GF, τ_IFSS_ > τ_AMS_) and high-temperature (Classes 2 and 8, 60 °C all fibers, τ_AMS_ ≪ τ_IFSS_) subcategories.

Classes 3 and 5, both involving polyester fiber, are classified as the mixed failure mode. This classification is supported by three mutually corroborating criteria that distinguish the mechanism from progressive pull-out or simple matrix failure accompanied by fiber elongation. In terms of the strength level, the τ_IFSS_ values of these two classes lie between those of the pure pull-out mode and the pure matrix failure mode at the same temperature, indicating that interfacial debonding and matrix deformation develop simultaneously rather than one being completely dominant. In terms of the curve morphology, the load–displacement curves combine the elastic ascending stage of interfacial bonding with a long plateau or gentle descending stage caused by fiber elongation, and the peak displacement is significantly larger than that of the brittle pull-out mode. In terms of the energy characteristic, the pull-out work of these classes is markedly higher than that of the pure matrix failure mode at the same temperature, reflecting the synergistic energy dissipation effect of interfacial debonding, matrix shear deformation, and fiber tensile elongation. Benefiting from the low modulus and high elongation at the break of the polyester fiber, multiple damage mechanisms act in concert during the pull-out process, forming a distinct mixed failure mechanism.

### 5.2. Coupled Influence of Fiber Type and Temperature on the Fiber and Asphalt Mastic Interfacial Failure

**Figure 15:**
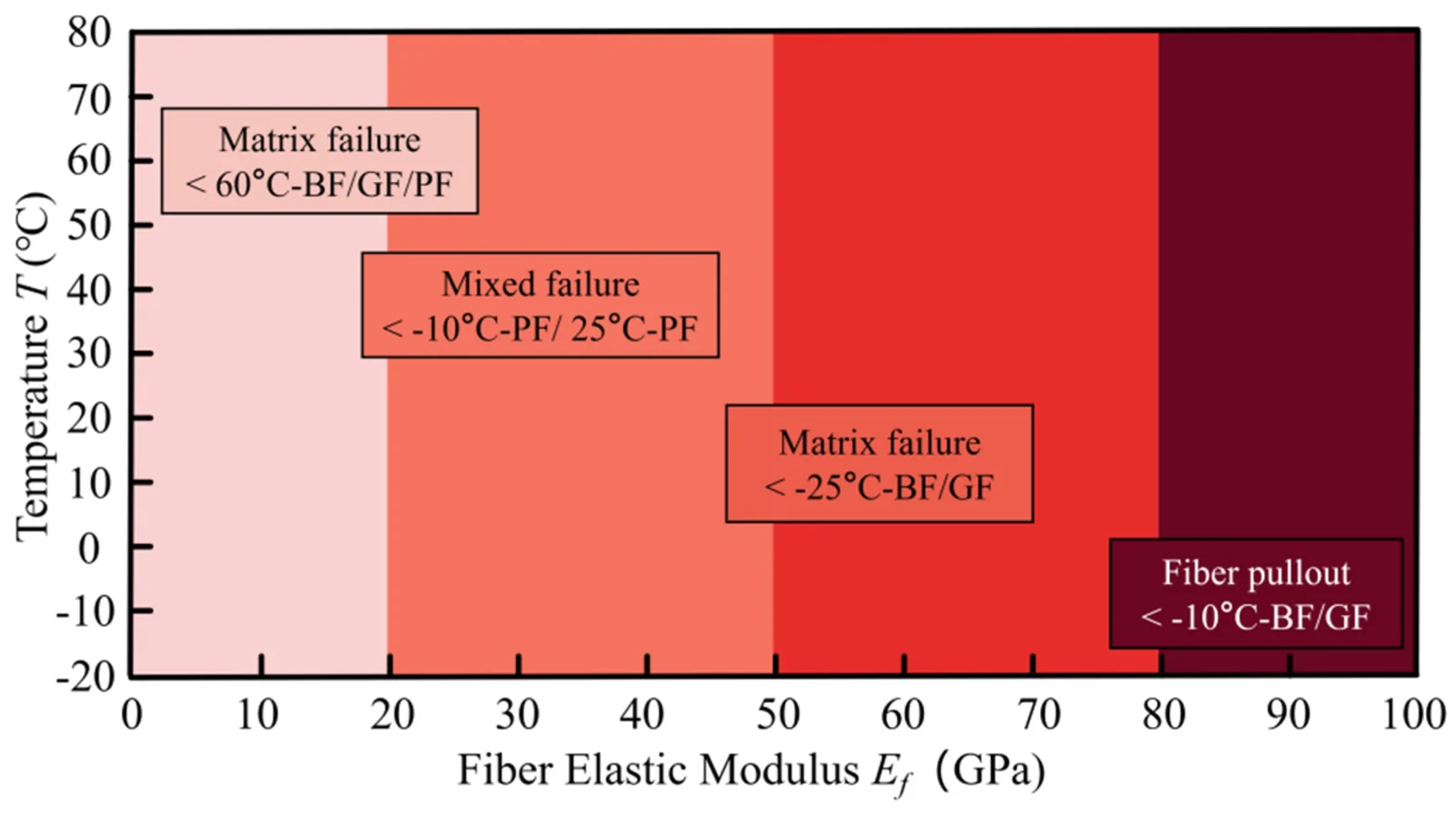
The interface failure rules affected by temperature and fibers.

Based on the physical mapping of eight clusters to failure modes, the coupled regulation mechanism of the temperature and fiber type on the interfacial failure behavior is systematically summarized, as shown in Figure 15.

Temperature dominates the preferential failure path by modulating the phase state and shear strength of asphalt mastic. As the temperature rises from −10 °C to 60 °C, asphalt mastic transitions from a glassy state through a viscoelastic state to a viscous flow state, with its shear strength decreasing by orders of magnitude. This shifts the weakest link of the system from the fiber–mastic interface to the mastic matrix, and the dominant failure mode evolves accordingly from interfacial pull-out at low temperature to matrix failure at high temperature. Fiber modulus governs the failure morphology and energy dissipation capacity by tuning the deformation compatibility between fiber and matrix. High-modulus basalt and glass fibers exhibit brittle failure characteristics with a steep post-peak load drop, while low-modulus polyester fiber forms a mixed failure mode combining progressive interfacial debonding, matrix shear deformation, and fiber elongation, resulting in markedly higher pull-out work.

**Figure 16:**
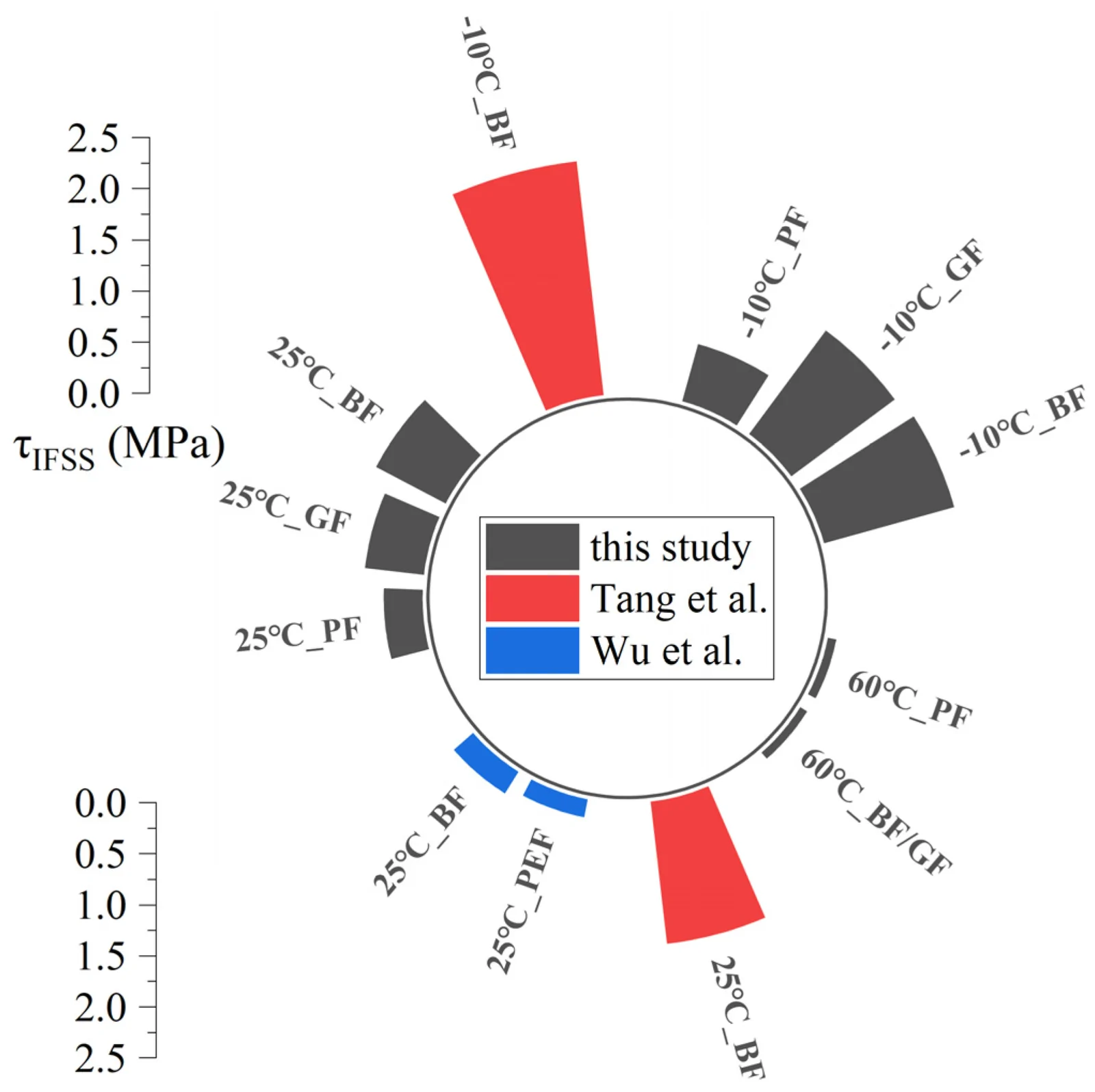
Comparison of different studies [12,50,51] with regard to asphalt mastic shear strength.

To verify the reliability of the experimental results, the interfacial shear strength values are compared with published single-fiber pull-out studies of similar systems [12,50,51], as illustrated in Figure 16. The τ_IFSS_ values obtained in this study range from 0.079 MPa at 60 °C to 1.32 MPa at −10 °C, which fall within the reasonable interval reported by the existing literature. All compared studies consistently show a sharp attenuation of interfacial strength with increasing temperature, and the strength ranking of basalt fiber > glass fiber > polyester fiber at the same temperature also agrees well with the previous findings. This study covers a wider temperature span across the glass transition region of asphalt mastic, complementing the systematic low-temperature interfacial strength data for fiber–mastic systems.

**Figure 17:**
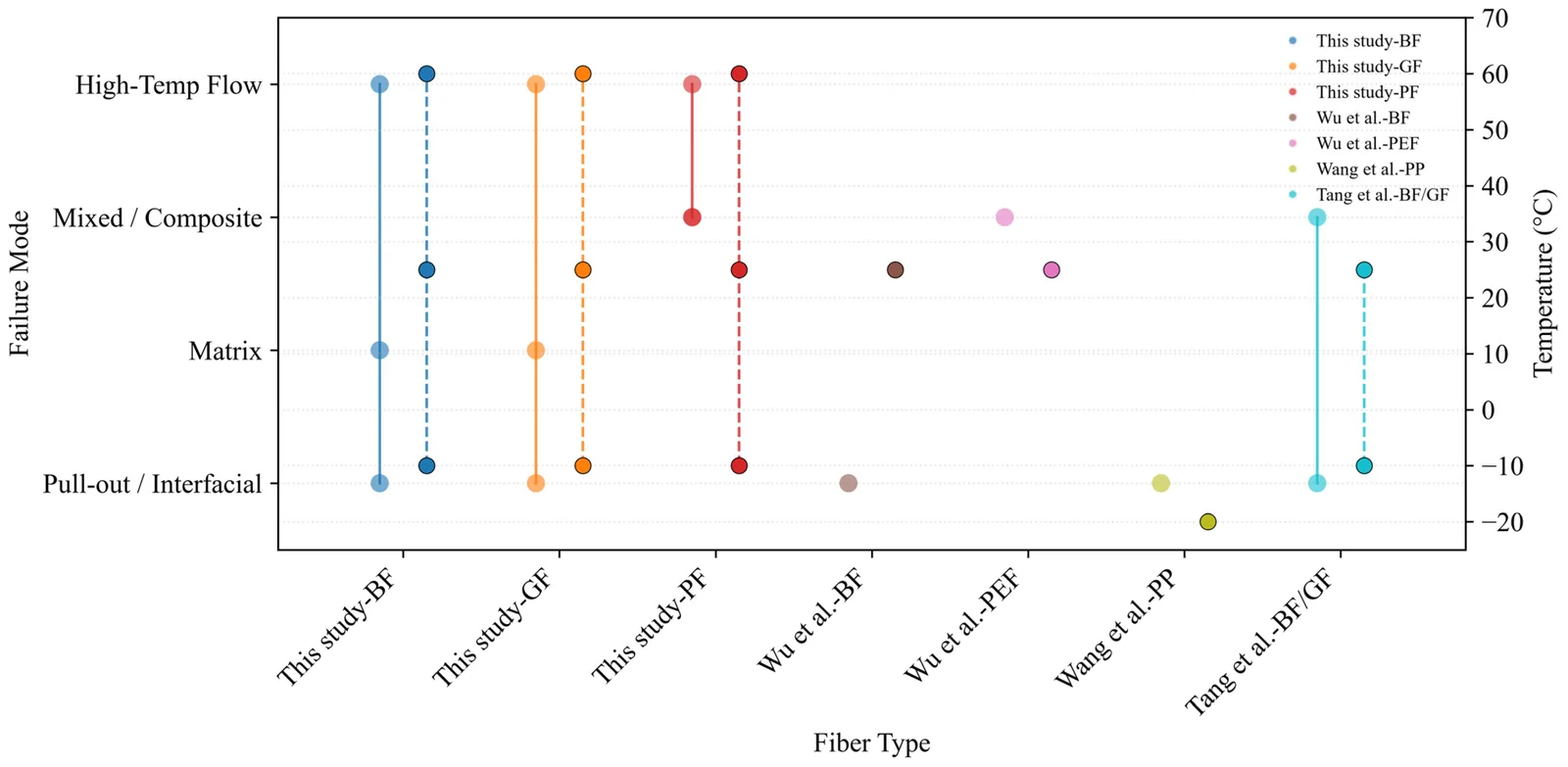
Comparison of failure modes of fiber extraction in different studies [12,50,51,52]. (Note: The solid lines represent the failure mode, while the dotted lines represent the temperature).

Figure 17 further compares the temperature-dependent evolution of failure modes across different studies [12,50,51,52]. A universal transition path is universally observed: interfacial pull-out dominates at low temperatures, mixed or composite failure occurs at medium temperatures, and viscous matrix failure prevails at high temperatures. The finding that high-modulus brittle fibers favor interfacial debonding while low-modulus ductile fibers tend to form mixed failure modes is highly consistent with previous experimental observations. Unlike conventional studies that rely on manual classification based on limited characteristic parameters, this study achieves quantitative failure mode identification through the unsupervised clustering of full load–displacement curves, improving the objectivity of mode discrimination.

Overall, the mixed failure mechanism of polyester fiber, which trades partial peak strength for a higher energy dissipation, shows potential advantages for low- and medium-temperature scenarios demanding both strength and toughness from a mesoscale interface perspective. This finding provides a reference for the fiber selection of reinforced asphalt materials, while its macro pavement performance requires further validation via asphalt mixture tests.

### 5.3. Performance Comparison Between the Improved Model and Conventional Methods

To demonstrate the advantages of the IGCMM for heterogeneous interfacial failure data, we compare it with four conventional approaches: (a) a single growth curve model, which assumes no mixture and treats all specimens as one class—this verifies the necessity of the mixture model; (b) a GCMM with a multivariate normal error distribution (shape parameter ***η*** = 1) in place of the MPE distribution—this validates the superiority of the MPE in fitting heavy-tailed data; (c) K-means clustering applied directly to the 200-dimensional raw load–displacement curves [53]; and (d) hierarchical clustering using Ward’s linkage on the raw high-dimensional curves [54]. The improved GCMM, the normal-error GCMM, and the single growth curve model were all trained in the low-dimensional principal component space (*k* = 3, cumulative explained variance 96.72%), whereas K-means and hierarchical clustering were conducted directly in the original 200-dimensional space. All methods used *r* = 8 classes to maintain a consistent baseline. The clustering performance was evaluated by the adjusted Rand index (ARI), using the IGCMM’s cluster labels as the reference. The goodness-of-fit metrics for each model are listed in Table 9.

As shown in the table, the single growth curve model yields a log-likelihood of −1383.81, indicating severe underfitting. This confirms the substantial heterogeneity of the interfacial failure behavior and justifies the need for a mixture model. The normal-error GCMM achieves a log-likelihood of −655.54, while the IGCMM attains −623.99, an improvement of approximately 4.8%. This validates the effectiveness of the MPE distribution in capturing the peaked and heavy-tailed residuals. The ARI values of both the IGCMM and the normal-error GCMM are 1.0, indicating that the distributional assumption has little impact on the clustering structure and that both models produce highly consistent class assignments. In contrast, K-means and hierarchical clustering conducted on raw 200-dimensional data yield an ARI of 0.7606 relative to the IGCMM partition, indicating an overall agreement of approximately 76% between the conventional methods and the proposed approach. When performed in the same three-dimensional principal component space as IGCMM, the ARI values increase slightly but remain below 0.85. This confirms that the advantage of IGCMM does not merely come from dimensionality reduction; more importantly, it introduces structured growth curve modeling for longitudinal data and probabilistic soft clustering, which can better capture the essential mechanical differences embedded in interfacial failure curves.

## 6. Conclusions

This study develops an improved growth curve mixture model (IGCMM) integrating PCA dimensionality reduction and the multivariate power exponential (MPE) distribution, and applies the model to identify the interfacial failure modes of fiber–asphalt mastic based on 90 single-fiber pull-out test datasets. The main findings are summarized as follows:

(1) The MPE distribution demonstrates a superior fitting performance for the peaked and heavy-tailed residuals of interfacial failure data, with a goodness-of-fit approximately 19.5% higher than the normal distribution (shape parameter *η* = 0.437 < 1). PCA compression from 200 dimensions to three principal components retains 96.72% of the total variance, reducing the covariance parameters from 20,100 to 6 and effectively avoiding the dimensionality challenge in high-dimensional model estimation.
(2) The IGCMM exhibits a favorable clustering stability and high classification certainty. The extended Bayesian information criterion (eBIC) selects eight optimal clusters. With multiple random initializations, the EM algorithm converges to a consistent solution. Posterior probability analysis shows that 96.7% of specimens have a class-membership probability exceeding 0.999, indicating a high internal consistency of the clustering results.
(3) The eight statistical clusters are inferred to correspond to four theoretical interfacial failure modes: fiber pull-out, intermediate-temperature matrix failure, high-temperature matrix failure, and mixed failure, as supported by comparisons of the interfacial shear strength, fiber fracture strength, and asphalt mastic shear strength.
(4) Temperature and fiber type exert a coupled regulatory effect on interfacial failure. Temperature dominates the failure path by altering the phase state of asphalt mastic—a low temperature tends to induce interfacial debonding, while intermediate and high temperatures mainly lead to matrix failure. The fiber modulus governs the curve morphology and energy dissipation: the pull-out work of the low-modulus high-ductility polyester fiber (E_f_ = 10.9 GPa) ranges from 0.724 to 1.156 mJ, higher than that of basalt and glass fibers (0.296–0.765 mJ) at the same temperature. From a mesoscale interfacial energy perspective, the mixed failure mechanism of polyester fiber, which trades some peak strength for a higher energy dissipation, may offer a reference for material selection in low- and intermediate-temperature applications, though this implication requires further validation through macro asphalt mixture performance tests.
(5) Future work may integrate scanning electron microscopy and digital image correlation to construct a multi-scale correlation model linking the cluster morphology, macro mechanical parameters, and micro damage evolution, to further clarify the physical mechanisms of interfacial failure.

## Figures and Tables

**Table 1:** Properties of each fiber.

Fiber Type	Diameter (μm)	Fracture Strength σ_FFS_ (MPa)	Elasticity Modulus E_f_ (GPa)
BF	16	2400	81.3
GF	16	2310	79.5
PF	16	870	10.9

**Table 2:** Data statistics results.

Temperature (°C)	Fiber	Peak Load Average Value (cN)	Peak Load CV (%)	The Mean Value of the Pull-Out Work (10^−2^ mJ)	The Pull-Out Work CV (%)
−10	BF	43.249	5.64	15.824	1.9
−10	GF	41.077	2.91	17.405	1.22
−10	PF	18.165	3.78	104.088	1.85
25	BF	24.189	4.74	54.301	0.44
25	GF	17.75	5.98	46.654	3.28
25	PF	11.98	3.58	68.657	1.13
60	BF	2.5	5.81	12.029	1.68
60	GF	2.444	6.98	10.28	3.09
60	PF	2.727	3.11	14.696	1.95

**Table 3:** Comparison of KS test results.

Distribution Pattern	KS Test Statistic D	KS Test *p*-Value	Goodness of Fit R^2^
Multivariate normal distribution	0.209221	<0.001	0.772245
Multivariate power exponential distribution	0.214779	<0.001	0.922512

**Table 4:** Reconstruction errors under different principal component numbers and the number of parameters.

The Number of Principal Components k	RMSE of the Reconfiguration	The Number of Parameters of the Covariance Matrix	The Total Number of Parameters
1	3.421	1	33
2	2.358	3	35
3	0.946	6	38
5	0.398	15	47
10	0.178	55	87
15	0.099	120	152
20	0.060	210	242
30	0.023	465	497

**Table 5:** The stability statistics results of 20 consecutive restarts.

Statistical Indicators	Value
Log likelihood—maximum	−623.9881
Log likelihood—minimum	−623.9881
Log likelihood—mean	−623.9881
Log likelihood—standard deviation	4.9 × 10^−12^
Average Adjusted RAND Index (ARI)	1.0000
The standard deviation of ARI	0.0000

**Table 6:** Estimation of regression coefficients for 8 types of damage patterns and the mixture ratio.

Class	β_k1_	β_k2_	β_k3_	Mixing Ratio π_k_
Class 1	−9.1808	9.4717	−1.3909	0.1111
Class 2	−0.3031	1.3888	−0.1761	0.1111
Class 3	−3.906	4.9873	−0.474	0.1111
Class 4	13.4808	−16.3228	2.0473	0.1061
Class 5	−6.2263	7.1036	−0.6285	0.1111
Class 6	−7.5467	8.0743	−1.1713	0.1111
Class 7	13.5603	−16.5704	2.0698	0.1161
Class 8	0.002	1.007	−0.1473	0.2222

**Table 7:** Statistical analysis of the physical and mechanical parameters of the eight types of clustering results.

Class	The Quantity of Samples	Operating Conditions	F_max_/cN	s_max_/mm	W_pull_/10^−2^ mJ	τ_IFSS_/MPa
1	10	25 °C-BF	23.21	1.15	76.5	0.77
2	10	60 °C-PF	2.60	2.50	12.6	0.086
3	10	25 °C-PF	11.57	5.50	72.4	0.38
4	9	−10 °C-BF	39.66	0.70	29.6	1.32
5	10	−10 °C-PF	17.56	8.45	115.6	0.58
6	10	25 °C-GF	17.38	1.25	64.5	0.58
7	11	−10 °C-GF	37.84	0.65	34.5	1.26
8	20	60 °C-BF/GF	2.39	1.10	11.9	0.079

**Table 8:** The summary of the physical mapping of the eight-category clustering results to the theoretical destruction patterns.

Failure Mode	Corresponding Category	The Quantity of Categories	The Quantity of Samples	Proportion	Corresponding Operating Conditions
Fiber pull-out	4, 7	3	20	22.2%	−10 °C-BF/GF
Matrix failure	1, 6	3	20	22.2%	25 °C-BF/GF
Matrix failure	2, 8	2	30	33.3%	60 °C-BF/GF/PF
Mixed failure	3, 5	2	20	22.2%	−10 °C-PF, 25 °C-PF

**Table 9:** Comparison of performance indicators for different methods.

Methods	Input Space	Log Likelihood	AIC	BIC	ARI	Numerical Anomaly
IGCMM	3 PCs	−623.99	1340.15	1455.14	1	FALSE
Single growth curve model	3 PCs	−1383.81	2789.61	2817.11	/	FALSE
Multivariate normal distribution-based GCMM	3 PCs	−655.54	1403.09	1518.08	1	FALSE
K-means	200-d raw data	/	/	/	0.7606	FALSE
K-means	3 PCs	/	/	/	0.8362	FALSE
Hierarchical clustering	200-d raw data	/	/	/	0.7606	FALSE
Hierarchical clustering	3 PCs	/	/	/	0.8137	FALSE

## Data Availability

The original contributions presented in this study are included in the article/Supplementary Materials. Further inquiries can be directed to the corresponding author.

## Supplementary Materials

The following supporting information can be downloaded at: https://www.mdpi.com/article/10.3390/ma19173615/s1, File S1: theorem proving; File S2: posterior_probabilities.

## Abbreviations

The following abbreviations are frequently used in this manuscript:

IGCMM	Improved growth curve mixture model
BF	Basalt fiber
GF	Glass fiber
PF	Polyester fiber
EM	Expectation–Maximization
MPE	Multivariate Power Exponential
eBIC	extended Bayesian Information Criterion
ARI	Adjusted Rand Index
IRLS	Iteratively Reweighted Least Squares
PCA	Principal Component Analysis
IFSS	Interfacial Shear Strength
