# Supplementary material for: Identification of Fiber and Asphalt Mastic Interface Failure Modes Based on the Improved Growth Curve Mixture Model
Source: Materials (Basel). 2026 Aug 25;19(17):3615. doi: 10.3390/ma19173615 (PMC13566478; doi:10.3390/ma19173615)
Supplement: Supplementary file 1 [file materials-19-03615-s001.zip › File S1. theorem proving.pdf]

Proof of Theorem 1 (Section 2.3.1)

Theorem Statement

Consider the growth curve model (GCM) defined as:

$$\begin{cases} Y_{p\times n}=X_{p\times l}B_{l\times r}Z_{r\times n}+\mathscr{E}_{p\times n} \\ \varepsilon\sim MPE_{p,n}(0,\Sigma,I_n,\boldsymbol{\beta}) \end{cases}$$

where the design matrix X and group matrix Z are of full column rank, $n>p+r$, $\Sigma>0$ is a positive definite covariance matrix, and $\boldsymbol{\beta}>0$ is the kurtosis parameter. Then the maximum likelihood estimators (MLEs) of the unknown parameters $\Theta=(B,\Sigma,\boldsymbol{\beta})$ exist and are unique. Specifically, the estimators of the regression coefficient matrix and covariance matrix satisfy:

$$\widehat{B}=\left(X'\Sigma^{-1}X\right)^{-1}X'\Sigma^{-1}YZ'\left(ZZ'\right)^{-1}$$

$$\widehat{\Sigma}=\frac{\boldsymbol{\beta}}{n}\left[tr\left((Y-X\widehat{B}Z)'\Sigma^{-1}(Y-X\widehat{B}Z)\right)\right]^{\boldsymbol{\beta}-1}(Y-X\widehat{B}Z)(Y-X\widehat{B}Z)'$$

The kurtosis parameter $\boldsymbol{\beta}$ can be solved via the Newton-Raphson iterative algorithm.

Proof

By the definition of the multivariate power exponential (MPE) distribution for matrix variates, the probability density function of the response matrix Y is given by:

$$f(Y;B,\Sigma,\boldsymbol{\beta})=K|\Sigma|^{-n/2}exp\left\{-\frac{1}{2}\left[tr\left((Y-XBZ)'\Sigma^{-1}(Y-XBZ)\right)\right]^{\boldsymbol{\beta}}\right\}$$

with the normalization constant:

$$K=\frac{pn\Gamma(pn/2)}{\pi^{pn/2}\Gamma(1+pn/(2\boldsymbol{\beta}))2^{1+pn/(2\boldsymbol{\beta})}}$$

Taking the natural logarithm of the density function yields the log-likelihood function:

$$\begin{aligned} l(B,\Sigma,\boldsymbol{\beta}) &= \ln(pn)+\ln\Gamma\left(\frac{pn}{2}\right)-\frac{pn}{2}\ln\pi-\ln\Gamma\left(1+\frac{pn}{2\boldsymbol{\beta}}\right)-\left(1+\frac{pn}{2\boldsymbol{\beta}}\right)\ln 2- \\ &\frac{n}{2}\ln|\Sigma|-\frac{1}{2}\left\{tr\left[(Y-XBZ)'\Sigma^{-1}(Y-XBZ)\right]\right\}^{\boldsymbol{\beta}} \end{aligned}$$

Step 1: MLE of the regression coefficient matrix B

Taking the matrix partial derivative of the log-likelihood with respect to B, and applying the chain rule together with the trace derivative rule $\frac{\partial tr(ABC)}{\partial B}=A'C'$,

$$\begin{aligned} \frac{\partial l}{\partial B} &=-\frac{1}{2}\cdot\boldsymbol{\beta}\cdot\left[tr\left((Y-XBZ)'\Sigma^{-1}(Y-XBZ)\right)\right]^{\boldsymbol{\beta}-1}\cdot\frac{\partial tr(\cdot)}{\partial B} \\ &=\boldsymbol{\beta}\cdot\left[tr\left((Y-XBZ)'\Sigma^{-1}(Y-XBZ)\right)\right]^{\boldsymbol{\beta}-1}\cdot vec\left(X'\Sigma^{-1}(Y-XBZ)Z'\right) \end{aligned}$$

Setting the partial derivative to zero, since $\boldsymbol{\beta}>0$ and the power term of the Mahalanobis trace is strictly positive, the normal equation reduces to:

$$X'\Sigma^{-1}(Y-XBZ)Z'=0$$

Rearranging terms gives:

$$X'\Sigma^{-1}XBZZ'=X'\Sigma^{-1}YZ'$$

By the full column rank assumption, $X'\Sigma^{-1}X$ and ZZ' are both invertible. Hence the MLE of B exists and is unique:

$$\widehat{B}=\left(X'\Sigma^{-1}X\right)^{-1}X'\Sigma^{-1}YZ'\left(ZZ'\right)^{-1}$$

Step 2: MLE of the covariance matrix Σ Taking the partial derivative of the log-likelihood with respect to Σ^{-1}, and using the determinant derivative rule $\frac{\partial \ln|\Sigma^{-1}|}{\partial \Sigma^{-1}}=\Sigma$, we have:

$$\frac{\partial l}{\partial \Sigma^{-1}}=\frac{n}{2}\Sigma-\frac{1}{2}\cdot\boldsymbol{\beta}\cdot\left[tr\left((Y-XBZ)'\Sigma^{-1}(Y-XBZ)\right)\right]^{\boldsymbol{\beta}-1}\cdot(Y-XBZ)(Y-XBZ)'$$

Setting the derivative to zero and multiplying both sides by 2:

$$n\Sigma=\boldsymbol{\beta}\cdot\left[tr\left(\left(Y-X\widehat{B}Z\right)'\Sigma^{-1}\left(Y-X\widehat{B}Z\right)\right)\right]^{\boldsymbol{\beta}-1}\cdot\left(Y-X\widehat{B}Z\right)\left(Y-X\widehat{B}Z\right)'$$

Dividing both sides by n yields the estimator of Σ:

$$\widehat{\Sigma}=\frac{\boldsymbol{\beta}}{n}\left[tr\left((Y-X\widehat{B}Z)'\Sigma^{-1}(Y-X\widehat{B}Z)\right)\right]^{\boldsymbol{\beta}-1}(Y-X\widehat{B}Z)(Y-X\widehat{B}Z)'$$

Step 3: Solution of the kurtosis parameter $\boldsymbol{\beta}$

Substituting $\widehat{B}$ and $\widehat{\Sigma}$ into the log-likelihood function yields a univariate function of $\boldsymbol{\beta}$. The first- and second-order derivatives with respect to $\boldsymbol{\beta}$ are derived, and the root of the score equation is obtained via the Newton-Raphson iteration:

$$\boldsymbol{\beta}^{(t+1)}=\boldsymbol{\beta}^{(t)}-\left(\frac{\partial^2 l}{\partial \boldsymbol{\beta}^2}\right)^{-1}\cdot\frac{\partial l}{\partial \boldsymbol{\beta}}|_{\boldsymbol{\beta}=\boldsymbol{\beta}^{(t)}}$$

Since the log-likelihood is unimodal in $\boldsymbol{\beta}$, the iteration converges to the unique global maximum. In summary, the MLEs of B, Σ and $\boldsymbol{\beta}$ exist and are unique. This completes the proof.

Proof of Statistical Properties of Generalized Least Squares Estimator (Section 2.3.3)

This subsection proves the unbiasedness of the generalized least squares estimator (GLSE) under the MPE distribution and derives its covariance structure, which provides the theoretical basis for initial value construction and clustering accuracy analysis.

Property Statement

Let $\widehat{B}=(X'X)^{-1}X'YZ'(ZZ')^{-1}$ be the GLSE of the regression coefficient matrix. Then:

1. Unbiasedness: $E(\widehat{B})=B$;
2. Covariance structure:

$$Cov\left(vec\left(\widehat{B}'\right)\right)=\frac{2^{1/\boldsymbol{\beta}}\Gamma\left(\frac{p+2}{2\boldsymbol{\beta}}\right)}{pn\Gamma\left(\frac{pn}{2\boldsymbol{\beta}}\right)}\cdot(X'X)^{-1}X'\Sigma X(X'X)^{-1}\otimes(ZZ')^{-1}$$

Proof

1. Proof of Unbiasedness

Substituting the model equation $Y=XBZ+\varepsilon$ into the GLSE expression, by the linearity of expectation:

$$\begin{aligned} E(\widehat{B}) &=(X'X)^{-1}X'E(Y)Z'(ZZ')^{-1} \\ =(X'X)^{-1}X'(XBZ)Z'(ZZ')^{-1} &=(X'X)^{-1}(X'X)B(ZZ')(ZZ')^{-1}=B \end{aligned}$$

The unbiasedness is proved.

2. Derivation of Covariance Structure

By the property of Kronecker product and vectorization operator $vec(ABC)=\left(C'\otimes A\right)vec(B)$,we

vectorize the transpose of $\widehat{B}$:

$$\begin{aligned} vec\left(\widehat{B}'\right) &= vec\left((ZZ')^{-1}ZY'X(X'X)^{-1}\right) \\ &= \left[\left(X(X'X)^{-1}\right)' \otimes (ZZ')^{-1}Z\right] vec(Y') \end{aligned}$$

By the property of the MPE distribution, $vec(Y')\sim PE_{pn}(vec((XBZ)'),\Sigma\otimes I_n,\boldsymbol{\beta})$,with covariance matrix:

$$Cov\left(vec(Y')\right)=\frac{2^{1/\boldsymbol{\beta}}\Gamma\left(\frac{p+2}{2\boldsymbol{\beta}}\right)}{pn\Gamma\left(\frac{pn}{2\boldsymbol{\beta}}\right)}\cdot(\Sigma\otimes\boldsymbol{I}_n)$$

Applying the linear transformation property of covariance:

$$\begin{aligned} Cov\left(vec\left(\widehat{B}'\right)\right) &= \left[(X'X)^{-1}X'\otimes(ZZ')^{-1}Z\right] Cov\left(vec(Y')\right) \left[X(X'X)^{-1} \otimes Z'(ZZ')^{-1}\right] \\ &= \frac{2^{1/\boldsymbol{\beta}}\Gamma\left(\frac{p+2}{2\boldsymbol{\beta}}\right)}{pn\Gamma\left(\frac{pn}{2\boldsymbol{\beta}}\right)}\cdot\left[(X'X)^{-1}X'\Sigma X(X'X)^{-1}\right]\otimes\left[(ZZ')^{-1}ZZ'(ZZ')^{-1}\right] \\ &= \frac{2^{1/\boldsymbol{\beta}}\Gamma\left(\frac{p+2}{2\boldsymbol{\beta}}\right)}{pn\Gamma\left(\frac{pn}{2\boldsymbol{\beta}}\right)}\cdot(X'X)^{-1}X'\Sigma X(X'X)^{-1}\otimes(ZZ')^{-1} \end{aligned}$$

The covariance structure is derived.

Degeneration Consistency Verification

When $\boldsymbol{\beta}=1$, by the recurrence relation of the gamma function $\Gamma(1+x)=x\Gamma(x)$, the coefficient term reduces to 1. The covariance structure then degenerates to the classical form of GLSE under the multivariate normal distribution, which is consistent with existing results and verifies the self-consistency of the model.

Proof of Theorem 2 (Section 2.4.2)

Theorem Statement

For the mixed growth curve model with unknown group matrix, let the group indicator vector $Z_{.i}\sim Cat(\pi_1,\ldots,\pi_r)$, and the error term follow the MPE distribution with kurtosis parameter $\boldsymbol{\beta}\in(0,1)$ (leptokurtic and heavy-tailed scenario). Then the parameter iterative updates based on the EM-MM algorithm satisfy:

1. The constructed surrogate function $Q\left(\Theta|\Theta^{(t)}\right)$ strictly minorizes the conditional expectation of the complete-data log-likelihood;
2. The iterative sequence monotonically increases the observed-data log-likelihood and converges to a local maximum;
3. The closed-form update formulas for each parameter are as follows:

Posterior class probability: $\hat{z}_{ki}^{(t+1)}=\frac{\hat{\pi}_k^{(t)}\cdot f_{PE}\left(y_i|X\hat{b}_k^{(t)},\hat{\Sigma}^{(t)},\boldsymbol{\beta}^{(t)}\right)}{\sum_{\mathrm{h}=1}^{r}\hat{\pi}_{\mathrm{h}}^{(t)}\cdot f_{PE}\left(y_i|X\hat{b}_{\mathrm{h}}^{(t)},\hat{\Sigma}^{(t)},\boldsymbol{\beta}^{(t)}\right)}$

Class prior probability: $\hat{\pi}_k^{(t+1)}=\frac{1}{n}\sum_{i=1}^{n}\hat{z}_{ki}^{(t+1)}$

Regression coefficient: $\hat{b}_k^{(t+1)}=\left(X'\left(\hat{\Sigma}^{(t)}\right)^{-1}X\right)^{-1}X'\left(\hat{\Sigma}^{(t)}\right)^{-1}\cdot\frac{\sum_{i=1}^{n}y_i\cdot\left(\hat{z}_{ki}^{(t+1)}\right)^{\boldsymbol{\beta}}\cdot\delta_{ki}^{(t)\boldsymbol{\beta}-1}}{\sum_{i=1}^{n}\left(\hat{z}_{ki}^{(t+1)}\right)^{\boldsymbol{\beta}}\cdot\delta_{ki}^{(t)\boldsymbol{\beta}-1}}$, where

$\delta_{ki}^{(t)}=\left(y_i-X\hat{b}_k^{(t)}\right)'\left(\hat{\Sigma}^{(t)}\right)^{-1}\left(y_i-X\hat{b}_k^{(t)}\right)$

Covariance matrix: $\hat{\Sigma}^{(t+1)}=\frac{\beta^{(t+1)}}{n}\sum_{i=1}^{n}\left[tr\left(\left(\hat{\Sigma}^{(t)}\right)^{-1}S_i^{(t+1)}\right)\right]^{\beta^{(t)}-1}S_i^{(t+1)}$, where $S_i^{(t+1)}=\sum_{k=1}^{r}\left(y_i-X\hat{b}_k\right)\left(y_i-X\hat{b}_k\right)'\hat{z}_{ki}^{(t+1)}$

Proof

Step 1: Complete-data Log-likelihood and E-step Derivation

The complete-data log-likelihood (including the latent group indicator Z) can be decomposed by sample:

$$\begin{aligned} l(\Theta;Y,Z) &= \sum_{i=1}^{n}\left[C-\ln\Gamma\left(1+\frac{p}{2\beta}\right)-\left(1+\frac{p}{2\beta}\right)\ln 2+\frac{1}{2}\ln|\Sigma^{-1}|\right] \\ &-\frac{1}{2}\sum_{i=1}^{n}\sum_{k=1}^{r}z_{ki}\cdot\left[\left(y_i-Xb_k\right)'\Sigma^{-1}\left(y_i-Xb_k\right)\right]^{\beta}+\sum_{i=1}^{n}\sum_{k=1}^{r}z_{ki}\ln\pi_k \end{aligned}$$

where $C=\ln(p\Gamma(p/2))-(p/2)\ln\pi$, is a constant independent of parameters.

In the E-step, given the current iterate $\Theta^{(t)}$ and observed data Y, we take the conditional expectation of the complete-data log-likelihood. By Bayes' theorem, the posterior probability that the i-th individual belongs to the k-th class is:

$$\hat{z}_{ki}^{(t+1)}=E\left(z_{ki}|y_i,\Theta^{(t)}\right)=\frac{\hat{\pi}_k^{(t)}\cdot f_{PE}\left(y_i|X\hat{b}_k^{(t)},\hat{\Sigma}^{(t)},\beta^{(t)}\right)}{\sum_{\mathrm{h}=1}^{r}\hat{\pi}_{\mathrm{h}}^{(t)}\cdot f_{PE}\left(y_i|X\hat{b}_{\mathrm{h}}^{(t)},\hat{\Sigma}^{(t)},\beta^{(t)}\right)}$$

This is the update formula for the posterior class probability.

Step 2: Proof of Minorization Property of Surrogate Function

When $\beta\in(0,1)$, the function $\varphi(x)=x^{\beta}$ is concave. Direct optimization of the power term in the conditional expectation is analytically intractable, so we introduce the Minorization-Maximization (MM) algorithm to construct a solvable surrogate function.

By the discrete form of Jensen's inequality: for a concave function $\varphi(\cdot)$, for any weights $\alpha_k\geq 0$ with $\sum_{k=1}^{r}\alpha_k=1$,

$$\varphi\left(\sum_{k=1}^{n}\alpha_k x_k\right)\geq\sum_{k=1}^{n}\alpha_k\,\varphi(x_k)$$

Let the Mahalanobis distance of individual i in class k be $f_k=\left(y_i-Xb_k\right)'\Sigma^{-1}\left(y_i-Xb_k\right)$. Define the weights:

$$w_k^{(t)}=\frac{\hat{z}_{ki}^{(t)}f_k^{(t)}}{\sum_{\mathrm{h}=1}^{r}\hat{z}_{\mathrm{h}i}^{(t)}f_{\mathrm{h}}^{(t)}}$$

which satisfy $\sum_{k=1}^{r}w_k^{(t)}=1$. Applying Jensen's inequality yields:

$$\left(E[f_k|y_i,\Theta^{(t)}]\right)^{\beta}\geq E\left(f_k^{\beta}|y_i,\Theta^{(t)}\right)$$

We then construct the surrogate function:

$$Q_1\left(\Theta|\Theta^{(t)}\right)=\sum_{i=1}^{n}constantterms-\frac{1}{2}\sum_{i=1}^{n}\left[tr\left(\Sigma^{-1}S_i^{(t+1)}\right)\right]^{\beta}+\sum_{i=1}^{n}\sum_{k=1}^{r}\hat{z}_{ki}^{(t+1)}\ln\pi_k$$

where $S_i^{(t+1)}=\sum_{k=1}^{r}\left(y_i-Xb_k\right)\left(y_i-Xb_k\right)'\hat{z}_{ki}^{(t+1)}$.

This surrogate function satisfies the two core conditions of the MM algorithm:
1. Minorization:$Q_1\left(\Theta|\Theta^{(t)}\right) \le E\left(l(\Theta)|Y,\Theta^{(t)}\right) for all \Theta$;
2. Tightness: Equality holds at the current iterate, i.e., $Q_1\left(\Theta^{(t)}|\Theta^{(t)}\right)=E\left(l\left(\Theta^{(t)}\right)|Y,\Theta^{(t)}\right)$.
Therefore, maximizing the surrogate function guarantees a non-decreasing observed-data log-likelihood, and the iteration is convergent.
Step 3: Closed-form Updates in M-step
Taking partial derivatives of the surrogate function with respect to π_k, b_k, Σ and $\boldsymbol{\beta}$, respectively, and setting them to zero, we derive the update formulas for each parameter:

1. Class prior probability π_k
Introducing the Lagrange multiplier λ for the constraint $\sum_{k=1}^{r} \pi_k = 1$, the solution is: $\hat{\pi}_k^{(t+1)} = \frac{1}{n} \sum_{i=1}^{n} \hat{z}_{ki}^{(t+1)}$

2. Regression coefficient b_k
Taking the partial derivative with respect to b_k and setting it to zero yields a weighted normal equation, whose solution is the weighted least squares form given in the theorem.
3. Covariance matrix Σ

Since $\left[tr\left(\Sigma^{-1} S_i\right)\right]^{\boldsymbol{\beta}}$ is still concave in Σ^{-1}, we construct a second-level surrogate function via the supporting hyperplane inequality. Taking the derivative yields the iterative update formula for Σ.
4. Kurtosis parameter $\boldsymbol{\beta}$
Substituting the updated values of other parameters into the surrogate function gives a univariate function of $\boldsymbol{\beta}$, whose optimum is solved via the Newton-Raphson iteration.

In summary, the EM-MM iteration satisfies the monotone ascent property of the likelihood and converges to a local maximum of the observed-data log-likelihood. This completes the proof.

Proof of Theorem 4 (Section 2.5.2)

Theorem Statement
For the mixed growth curve model with unknown group matrix and kurtosis parameter $\boldsymbol{\beta} \in [1,+\infty)$(platykurtic scenario), perform the Jordan spectral decomposition of the covariance matrix:$\Sigma^{-1} = D\Lambda^{1/\boldsymbol{\beta}} D'$, where D an orthogonal matrix and Λ is a diagonal matrix of eigenvalues. Then:
1. The eigenvalue matrix Λ has a closed-form iterative solution;
2. The orthogonal matrix D can be solved via gradient descent on the Stiefel manifold, and the Armijo line search guarantees convergence to a local optimum;
3. When $\boldsymbol{\beta}=1$, the estimation results degenerate to the corresponding form of the $\boldsymbol{\beta} \in (0,1)$ case, maintaining model consistency over the full parameter domain.
Proof
Step 1: Transformation of Objective Function via Spectral Decomposition
When $\boldsymbol{\beta} \ge 1$, $x^{\boldsymbol{\beta}}$ is a convex function, and the concave minorization technique in the previous MM algorithm is no longer applicable. We therefore decompose the high-dimensional matrix optimization problem into two low-dimensional subproblems — eigenvalue optimization and orthogonal matrix optimization — via spectral decomposition of the covariance matrix, and solve them by alternating iteration.

Substituting $\Sigma^{-1}=D\Lambda^{1/\beta}D'$ into the conditional expected log-likelihood, let $v_{ik}=D'\left(y_i-X\hat{b}_k\right)$ and $V_{ik}=v_{ik}v_{ik}'$. Then the power term of the Mahalanobis distance can be rewritten in trace form:

$$\left[\left(y_i-Xb_k\right)'\Sigma^{-1}\left(y_i-Xb_k\right)\right]^{\beta}=\left(\sum_{h=1}^{p}\lambda_h^{1/\beta}v_{ikh}^2\right)^{\beta}=tr\left(\Lambda^{1/\beta}V_{ik}\right)^{\beta}$$

The objective function now contains only two sets of unknowns: eigenvalues Λ and orthogonal matrix D. We fix one and optimize the other alternately until convergence.

Step 2: Closed-form Iterative Solution for Eigenvalue Matrix Λ

Fixing the orthogonal matrix D, the objective function remains concave in the eigenvalues $\lambda_1,\ldots,\lambda_p$. We construct a surrogate function using the supporting hyperplane inequality. Taking the first-order Taylor expansion of $f(\lambda)=tr\left(\Lambda^{1/\beta}V_{ik}\right)^{\beta}$ at the current iterate $\hat{\lambda}^{(t)}$(upper bound for concave functions):

$$f(\lambda)\leq f\left(\hat{\lambda}^{(t)}\right)+\nabla f\left(\hat{\lambda}^{(t)}\right)'\left(\lambda-\hat{\lambda}^{(t)}\right)$$

Substituting the surrogate function into the objective, taking the partial derivative with respect to Λ^{-1} and setting it to zero, we obtain the update formula for eigenvalues:

$$\left(\hat{\Lambda}^{-1}\right)^{(t+1)}=\frac{\hat{\beta}^{(t+1)}}{n}\sum_{i=1}^{n}\sum_{k=1}^{r}\hat{\pi}_k^{(t+1)}\cdot\left[tr\left(\left(\hat{\Lambda}^{(t)}\right)^{1/\hat{\beta}^{(t+1)}}V_{ik}^{(t+1)}\right)\right]^{\hat{\beta}^{(t+1)}-1}\cdot V_{ik}^{(t+1)}\left(\hat{\Lambda}^{(t)}\right)^{1/\hat{\beta}^{(t+1)}-1}$$

The eigenvalues have a unique closed-form iterative solution, which proves conclusion 1.

Step 3: Convergence Proof for Orthogonal Matrix Optimization

Fixing the eigenvalues Λ, the feasible domain of the orthogonal matrix D is the p-th order orthogonal Stiefel manifold:

$$\mathcal{M}=\left\{D\in\mathbb{R}^{p\times p}:D'D=I_p\right\}$$

The Riemannian gradient of the objective function on the manifold is the orthogonal projection of the Euclidean gradient onto the tangent space:

$$grad_{\mathcal{M}}f(D)=\nabla f(D)-D\cdot\frac{D'\nabla f(D)+\nabla f(D)'D}{2}$$

where the Euclidean gradient is $\nabla f(D)=\sum_{k=1}^{r}2\beta\left(Q_kD\Lambda^{-1/\beta}D'\right)^{\beta-1}Q_kD\Lambda^{-1/\beta}$, with $Q_k=\sum_{i=1}^{n}\hat{\pi}_k^{1/\beta}\left(y_i-Xb_k\right)\left(y_i-Xb_k\right)'$.

We use QR decomposition as the retraction mapping $R_D(V)=qf(D+V)$, i.e., the orthogonal factor in the QR decomposition of matrix $D+V$, combined with the Armijo inexact line search for step size selection. According to Riemannian optimization theory, the steepest descent algorithm with Armijo line search guarantees monotonic decrease of the objective function, and the iterative sequence converges to a critical point (local minimum) of the objective. This proves conclusion 2.

Step 4: Degeneration Consistency Verification

When $\beta=1,\Sigma^{-1}=D\Lambda D'$, reduces to the ordinary spectral decomposition. The power term of the Mahalanobis distance degenerates to a standard quadratic form. After simplification, the iterative formulas for eigenvalues and orthogonal matrix are fully consistent with the covariance estimator in the $\beta\in(0,1)$ case at $\beta=1$, ensuring the self-consistency of the model over the full parameter domain. This proves conclusion 3.

In summary, Theorem 4 holds.
